# Adsorption of Pb (II) ions on variable charge oxidic calcined substrates with chemically modified surface

**DOI:** 10.12688/f1000research.132880.1

**Published:** 2023-06-26

**Authors:** José G. Prato, Fernando Millán, Marialy Rangel, Andrés Márquez, Luisa Carolina González, Iván Ríos, César García, Carlos Rondón, Enju Wang

**Affiliations:** 1 Escuela de Ingeniería Química, Facultad de Ingeniería, Universidad de Los Andes, Mérida, 5101, Venezuela; 2 Grupo de Investigación Estudios Interdisciplinarios, Ingeniería Ambiental, Facultad de Ingeniería, Universidad Nacional de Chimborazo, Riobamba, Chimborazo Province, 060103, Ecuador; 3 Chemical Engineering School, Polytechnical Institute Santiago Mariño, IUPSM-Mérida, Mérida, 5101, Venezuela; 4 Facultad de Farmacia y Bioanálisis, Universidad de Los Andes, Mérida, 5101, Venezuela; 5 Grupo de Investigación “Análisis de Muestras Biológicas y Forenses”, Laboratorio Clínico, Facultad de Ciencias de la Salud, Universidad Nacional de Chimborazo, Riobamba, Chimborazo Province, 060103, Ecuador; 6 Arquitectura, Facultad de Ingeniería, Universidad Nacional de Chimborazo, Riobamba, Chimborazo Province, 060103, Ecuador; 7 Departamento de Química, Facultad de Ciencias, Universidad de Los Andes, Mérida, 5101, Venezuela; 8 Department of Chemistry, Saint John´s University, New York, Dodoma Region, Queens, USA

**Keywords:** ionic adsorption, calcined substrate, Pb(II) ions, isotherms

## Abstract

**Background:** The adsorption process is an alternative method for treating natural and waste waters, with heavy metals. Oxidic lithological materials, rich in iron and aluminum amphoteric oxides, with pH-dependent surface charges, are a reliable medium for ionic adsorption. Being thermally resistant, these materials can be used to prepare a calcined substrate which is chemically treated in an acid or alkaline solution to enlarge surface positive or negative charge density, making it possible anion as well as cation adsorption reactions from aqueous solutions. Oxidic lithological materials use is a low-cost alternative for filtering system because of its availability and ease of preparation and application.

**Methods:** Present paper shows results of the adsorption reaction of Pb ^+2^ ions on calcined substrates prepared with oxidic lithologic material. The study was performed on the substrate with chemically modified surface in alkaline medium as well as on non-treated surface.

**Results:** Results show *L*-type isotherms for the adsorption on the activated substrate, indicative affinity between adsorbate and adsorbent. Average value of adsorption capacity ( *k*) for activated substrate is around 3.7 times greater (1791.73±13.06) compared to the respective average *k* value for the non-activated substrate (491.54±31.97), during the adsorption reaction, 0.35 and 0.26 mmolH ^+^ of proton are produced on the activated and non-activated substrate respectively using a 1 *m*M Pb ^+2^ solution and 72.2 and 15.6 mmolH ^+^ using a 10 *m*M Pb ^+2^ solution. This acidification agrees with the theoretic model of transitional metals chemisorption on amphoteric oxides of Fe, Al, Ti and Mn present in lithological material used for the preparation of adsorbent substrates confirming the information given by the *L*-type isotherms.

**Conclusions:** Results suggest that these oxidic lithologic materials show great potential as an alternative technique for water treatment and heavy metal retention from contaminated waters using a low-cost and reliable adsorption system.

## Introduction

Lead (Pb) is a toxic element with the greatest global distribution among heavy metals, ^1^^–^^3^ it reaches water sources through various anthropogenic activities such as pipe corrosion, mining, oil refining, metal processing, glass production, battery manufacturing, among other industrial activities. Lead pipes were used regularly for water supply network until the seventies however, old constructions still remain and new residential buildings contain plumbing devices for water services. ^4^^–^^6^ Recently lead-contaminated water in some High Schools in Brooklyn, New York has been detected at high concentrations, exceeding the Environmental Protection Agency’s (EPA’s) action level of 0.015 mg/L. ^4^^,^^5^ Depending on the local water characteristics, such as pH, hardness and temperature, the lead in the pipes and faucets can dissolve, becoming a health risk for the consumers. ^6^ However, lead contamination is also the consequence of non-treated industrial wastes from paintings, batteries, ceramics factories, and mining activities. ^2^^,^^3^ Leaded fuels are still used in many South America countries with great impact to the environment. ^7^

The conventional methods for removing heavy metals from water and sewage have been well described in literature. ^1^^,^^2^^,^^8^^–^^10^ However, most of these industrial procedures are expensive and few supply companies can offer these systems. Some of them, such as precipitation, coagulation and sedimentation have disadvantages of generating sludge that requires further treatment, which increases operational costs. Non-conventional procedures have been investigated including the use of different types of biomass and organic adsorbents as agricultural by-products and its conversion to activated carbon, ^9^^,^^11^^,^^12^ biopolymers. ^9^^,^^13^ Some fungal biomasses have been used with 80 to 100 % percentage retention. ^14^ Natural and synthetic zeolites have been also used for lead removal from water showing great affinity and selectivity. ^15^^,^^16^ Amorphous nanoaluminophosphates have also been proved for lead removing from aqueous solution with efficiency between 40 and 70%, ^17^^,^^18^ used rice straw derived biochar as amendment in variable charge soils for Pb immobilization, avoiding its run off through the soil and protect the rivers. Although most of these methods may be efficient, they are expensive and/or difficult application at medium and large scale.

In a previous study ^19^^–^^21^ some oxidic lithological materials were described and characterized in order to use them for preparing a calcined substrate and apply it for ionic adsorption from aqueous solution. The high content of amphoteric iron and aluminium, as well as titanium and manganese oxides, with pH dependent variable charges surfaces. As a consequence of this particular property, these lithological materials are versatile for preparing calcined adsorbing substrates which have been applied in water softening, ^22^^–^^24^ water treatment and organic matter removing. ^25^^,^^26^ The study of copper and zinc adsorption on this kind of substrate revealed that these transitional metals participate in a specific chemisorption reaction which is pH dependent, especially copper ions. ^19^^,^^27^ The last study suggests that other transitional metals may participate in similar kind of specific adsorption reaction. By means of acid treatment of calcined substrate, adsorption of oxyanions as sulphate and phosphate adsorption have also been reported in the literature. ^21^^,^^28^^,^^29^ The main objective of the present work is to study the relative affinity between the oxidic calcined surface and Pb (II) ions, and its potentiality as water treatment system for lead ions removing.

## Methods

### Oxidic lithological material

The raw oxidic lithological material (OLM) was collected from a natural deposit, a mine, 5 kg were extracted manually with the help of pickaxes and shovels in the coodinates 8 ^o^ 28´47´´ N and 71 ^o^ 23´47´´ W. This zone is an arid type, where the temperature varies between 17 and 30 ^o^C along the year, and maximum rainfall of 200 mm per year. The OLM has already been chemically characterized, ^19^^–^^21^ consisting mainly of Fe, Al as well as Ti and Mn which can form refratory amphoteric oxides. Due to its thermal resistence this lithologic material is used by potters for preparing bricks by thermal treatment.

### Preparation of the adsorbent substrate and activation of negative surface charges

The lithologic material was crushing using a rubber hammer so as not to destroy the mineral structures, and sieved for 5 minutes, using Octagon 200CL Digital Sieve Shaker (Endecotts Ltd, England) to obtain particle-size fractions of 800 μm, then mixed up with distillated water in order to obtain a homogeneous saturated paste with an easily moldable consistency. With the use of a 60 mL syringe, 3 mm diameter “spaghettis” are extruded and cut out into 5 mm long pieces, are dried in ambient air for 24 hours, then in a furnace at 150 ^o^C another 24 hours (FELISA FE-293, Jalisco, Mexico). Finally, the dried solid adsorber is calcined up to 750 ^o^C at the rate of 6.25 ^o^C min ^-1^, across four hours (Thermolyne FB141OM muffle, Thermo Scientific, Waltham, USA) and then cooled down for 12 hours, until 20 ^o^C. The calcination process favors the oxides formation and cementation of the pellets to avoid dispersion in the aqueous solution. Also, during the calcination process, organic matter fraction, which is very small, is complete burned out, so only the oxidic phase participates in the adsorption reaction.

The calcined substrate is chemically treated in alkaline media with 0.1 N NaOH solution for 12 hours in a flask at room temperature, in order to enlarge the negative charge density on the surface because of the oxide deprotonation reaction. The product is then called activated substrate. (The non-treated substrate is labeled with the name of non-activated substrate). After this time the substrate is washed out with distilled water until neutral *p*H is achieved, then dried in the furnace at 120 ^o^C for 12 hours.

**Adsorption studies**

The adsorption study was performed in triplicate in isothermal conditions (20 ± 2 ^o^C) using batch equilibration procedure, treating 2 g of activated and non-activated calcined substrate, with increasing volume of 5, 10, 15, 20, 25, 30 and 40 mL of 0.001 M Pb ^+2^ solution, prepared from CH _3_COOPb analytical grade Merck reagent, during 24 hours. in 100 mL glass beakers. Suspensions were periodically shaken every hour. Differences between *Ci* and *C _eq_* were assumed to be due to adsorption. Adsorption isotherms were obtained by plotting the amount of lead adsorbed ( *q _e_*), against the Pb ^+2^ equilibrium concentration ( *C*_eq_) and fitted to the linear forms of the Freundlich and Langmuir equations. ^21^^,^^30^^–^^33^

Freundlich Isotherm is an empirical model which assumes an adsorption process characterized by multy layer adsorption on heterogeneous surfaces. The model is described by the equation 1 and the linear form by equation 2. ^21^^,^^30^^,^^31^ A graph representation of log (q _e_) vs log (C _eq_) is a straight line, with slope equal to n and intercept equal to log (K _F_):

$$q_{e}=K_{F}*C_{\mathrm{eq}}^{\left(n\right)}$$ (1)

$$\log\left(q_{e}\right)\;=\log\;\left(K_{F}\right)\;+\;n*\log\;\left(C_{\mathrm{eq}}\right)$$ (2)

In equations (1) and (2), q _e_ is the amount adsorbed per adsorbent weight unit, C _eq_ is the equilibrium concentration of adsorbate in solution after adsorption, K _F_ is the Freundlich constant related to adsorption capacity, and n is a constant related to adsorption intensity or energetic homogeneity of active sites of adsorption. n may take values near unity or greater. The lower the value of n is, the lower the energy heterogeneity in the active adsorption sites.

The Langmuir model describes a reversible process with the formation of adsorbate monolayers on the adsorbent surface. The nonlinear and linear forms of the Langmuir isotherm are described in Equations (3) and (4) ^21^^,^^22^^,^^33^:

$$q_{e}=\frac{q_{m}*K_{L}*C_{\mathrm{eq}}}{1+K_{L}*C_{\mathrm{eq}}}$$ (3)

$$\frac{1}{q_{e}}=\left[\frac{1}{K_{L}*q_{m}}\right]*\frac{1}{C_{\mathrm{eq}}}+\frac{1}{q_{m}}$$ (4)

In equations (3) and (4), q _m_ is the adsorption capacity, which represents the maximum amount of adsorbate in a monolayer, and K _L_ is the Langmuir constant related to the affinity between adsorbate and adsorbent. The parameters of the isotherm are obtained by plotting 1/q _e_ versus 1/C _eq_, resulting in a straight line with slope equal to 1/K _L_*q _m_ and the intersection equal to 1/q _m_.

The best fit between the isotherm function and the experimental data was verified by carrying out linear regressions of the isotherm linear equation. In addition, the adjustment of each isotherm was verified by comparing the experimental value obtained from the amount adsorbed (q _e_ exp) with the value obtained by calculating it by means of the isotherm equation (q _e_ calc) and determined the linear correlation between the two values given by the linear regression coefficient (r).

**Lead analysis**

Pb ^+2^ analyses were performed with a Varian Graphite Furnace Atomic Absorption Spectrophotometer, Spectra AA Zeeman 220, with a pyrolytic coated graphite tube (Palo Alto, California, USA). The spectrophotometer is dotted with a Varian auto sampler model EL-97113008. A Varian Uranium hollow cathode lamp was used.

***p*H and electric conductivity studies**

Solution *p*H and electric conductivity (EC) variations were studied, in triplicate, using the same isothermal batch equilibration procedure, as in adsorption study. *p*H was measured with a HI 211 *p*Hmeter (HANNA instruments, Smithfield, Rhode Island, USA), calibrated with commercial buffer solutions of *p*H 4 and 7. EC was measured with a Trans Instrument HC3010 Conductimeter (Petro Centre, Bukit Merah, Singapure), calibrated with standard reference. All experimental data ^34^ were worked out with the Excel software (Microsoft, Los Angeles, USA)

## Results

### Isotherm graph

**Figure 1.**
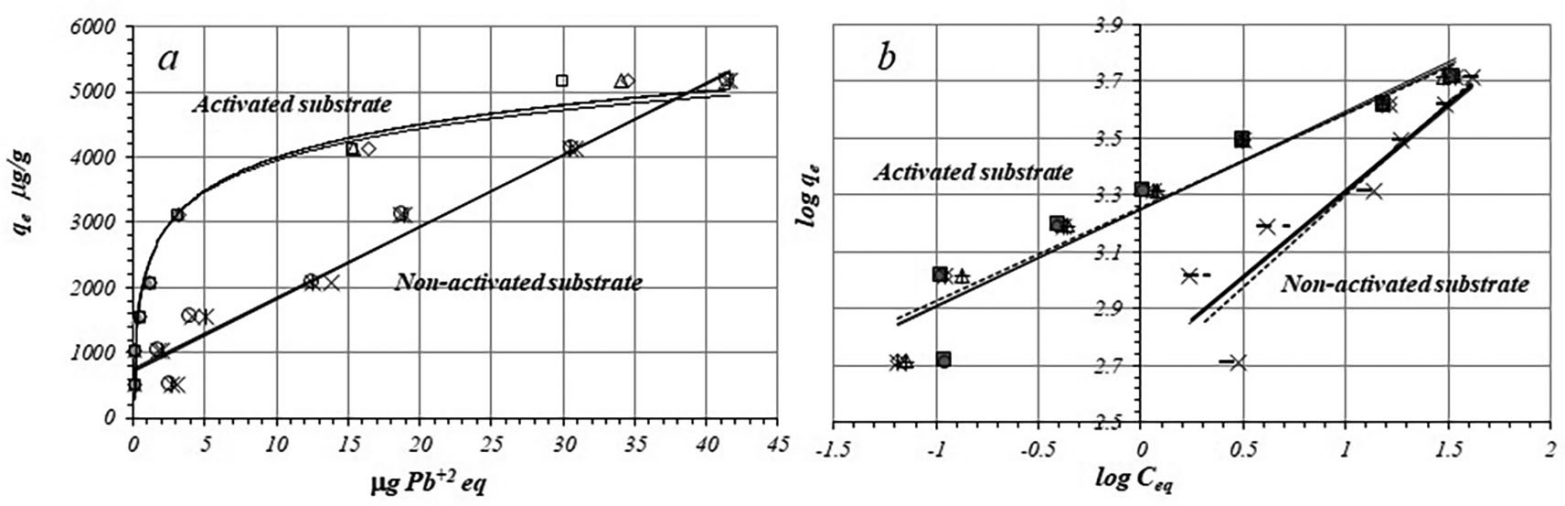
***a*:** Isotherms for Pb ^+2^ ions adsorption and ***b*:** fitting to the logarithmic form of the Freundlich model for the activated and non-activated substrates.

Figure 1a shows the adsorption isotherms of Pb ^+2^ ions on the activated and non-activated substrates. There are two relevant aspects to consider. First of all, the isotherm profiles show but don´t confirm information which kind of interaction between Pb ^+2^ ions and substrate surface may take place. In addition, the system under study coincides with a *L*-type isotherm, that is, at low equilibrium concentrations the amount adsorbed increases rapidly, while at high concentrations it decreases, due to the fewer adsorption sites available on the adsorbent].

Figure 1b shows linear fitting of adsorption data to the Freundlich model for the activated and non-activated substrates, while the Table 1 shows the fitted equations according to the Freundlich model, *r*, *K _F_*, and *n* values for activated and non-activated substrates, by triplicated trials. In both cases data present relatively good linearity, *r* being more favorable for the activated substrate, the negative charge density on the surface is likely to be more homogeny than in non-activate substrate.

**Table 1.** Fitted equations according to the Freundlich model, *r*, *K _F_*, and *n* values for activated and non-activated substrates, by triplicated.

Substrate	Fitted equation	*r*	*K _F_*	*n*
Activated	y=3.2523+0.3446x	0.9733	1787.72	2.9
	y=3.2568+0.3294x	0.9713	1806.34	3.0
	y=3.2507+0.3366x	0.9497	1781.15	3.0
Non-activated	y=2.7061+0.5998x	0.9098	508.28	1.7
	y=2.6577+0.6434x	0.9423	454.67	1.6
	y=2.7090+0.6052x	0.9268	511.68	1.7

Freundlich parameters are different for the activated substrate compared with the adsorption on the non-activated substrate. Average value of *K _F_*, for activated substrate is around 3.7 times greater (1791.73 ± 13.06) with a relative standard deviation (RSD) of 0.72 %, compared to the respective average *K _F_* value for the non-activated substrate (491.54 ± 31.97 with an RSD of 6.5 %). Similarly, the *n* values suggest greater adsorption intensity on the activated substrate in relation to the non-activated substrate. The smallest negative charge density on the non-activated substrate surface is a limiting factor for the adsorption of Pb ^+2^ ion.

**Figure 2.**
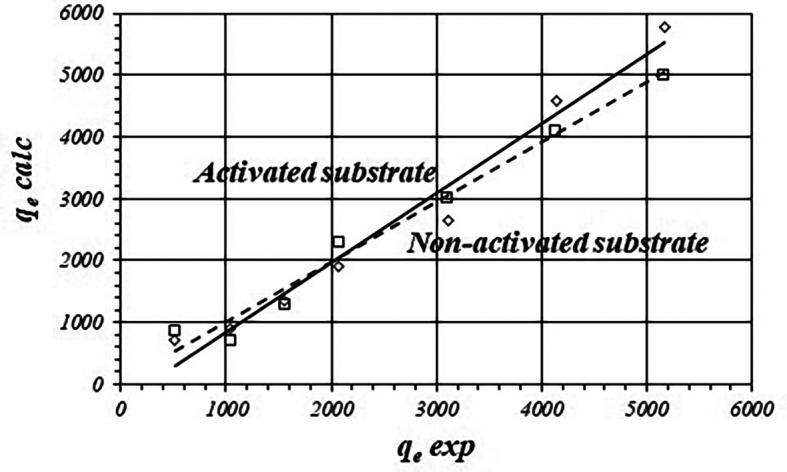
Correlations between calculated and experimental values of *q _e_* (Freundlich model) for the activated and non-activated substrates.

However, to prove adjustment to the model it is also necessary to show how good experimental data fit to the theoretic model. Figure 2 shows the graphical correlation between calculated and experimental *q _e_* data for the activated and non-activated substrates. Table 2 shows fitted equations for the linear functions. There is good linear correlation showing acceptable adjustment with the Freundlich model. However, calculated values of *q _e_* are biased from the experimental data. The average difference is about 15.16 ± 6.63 % in the case of the activated substrate and 17.41 ± 18.15 % for the non-activated substrate.

**Table 2.** Fitted equations for the correlation between *q _e_* calculated and *q _e_* experimental (Freundlich model).

Substrate	Fitted equation	*r*
Activated	y=−270.63+1.1222x	0.9858
Non-activated	y=44.206+0.9671x	0.9893

**Figure 3.**
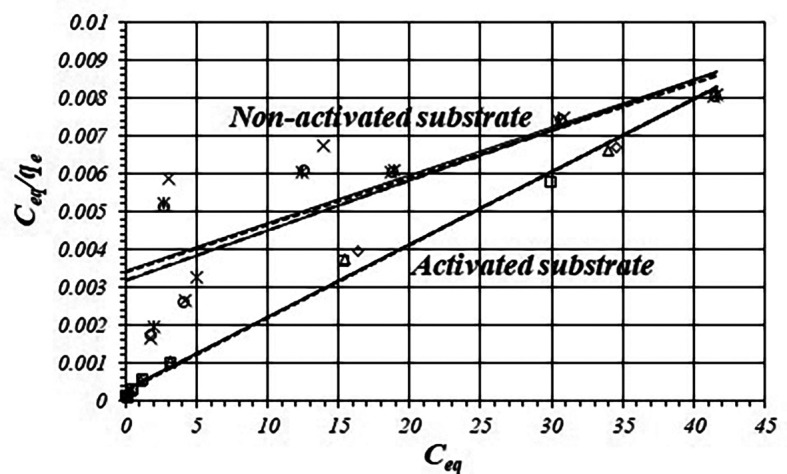
Fitting isotherms to the linear form of the Langmuir equation for the activated and non-activated substrates.

Figure 3 shows adsorption data fitting according to the Langmuir model and Table 3 shows fitted equations and values of *r*, *k*_1_ and *k*_2_ values for the activated and non-activated substrates, by triplicated. The experimental data shows great reproducibility, correlation coefficients are more satisfactory for the activated substrate in relation with the non-activated substrate. The *K _1_* value for the activated substrate is about 20 times greater than for the non-activated substrate.

**Table 3.** Fitted equations according to the Langmuir model, *r*, *k*_1_ and *k*_2_ values for activated and non-activated substrates, by triplicated.

Substrate	Fitted equation	*r*	*k _1_*	*k _2_*
Activated	y=0.0003+0.0002x	0.9937	0.6667	5000
	y=0.0003+0.0002x	0.9945	0.6667	5000
	y=0.0003+0.0002x	0.9950	0.6667	5000
Non-activated	y=0.0034+0.0001x	0.7993	0.0294	10000
	y=0.0034+0.0001x	0.8638	0.0294	10000
	y=0.0032+0.0001x	0.8578	0.0294	10000

**Table 4.** Correlations between calculated and experimental value of *q _e_* (Langmuir model) for the activated and non-activated substrates.

Substrate	Fitted equation	*r*
Activated	y=−365.81+1.0936x	0.9809
Non-activated	y=−221.28+1.1581x	0.9784

**Figure 4.**
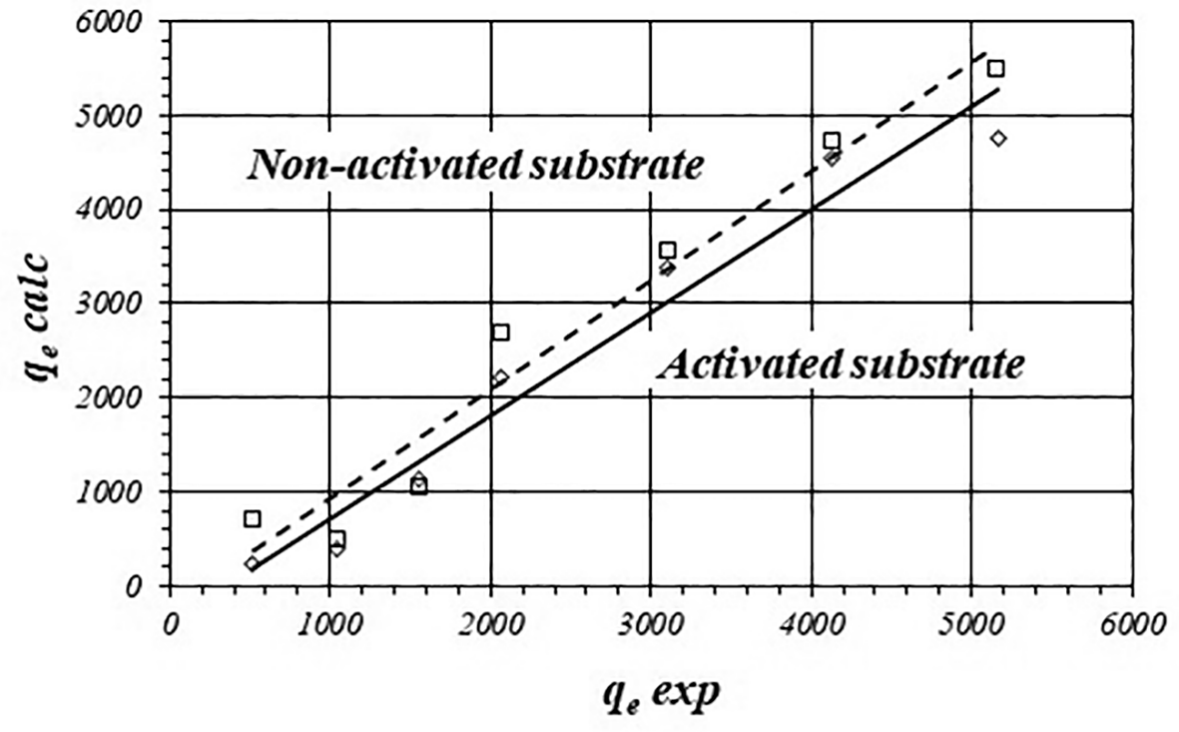
Correlations between calculated value of C _ad_ *vs* experimental value (Langmuir model) for the activated and non-activated substrates.

Figure 4 show the graphical correlations between calculated and experimental values of *q _e_* for the activated and non-activated substrates and Table 4 shows fitted equations for the linear functions. There is also good linear correlation between calculated and experimental data however, calculated values of *q _e_* are even more biased from the experimental data compared with the Freundlich model. The average difference is 25.29 ± 23.67 % in the case of the activated substrate and 27.22 ± 16.02 % for the non-activated substrate.

### *p*H and electric conductivity study

**Figure 5.**
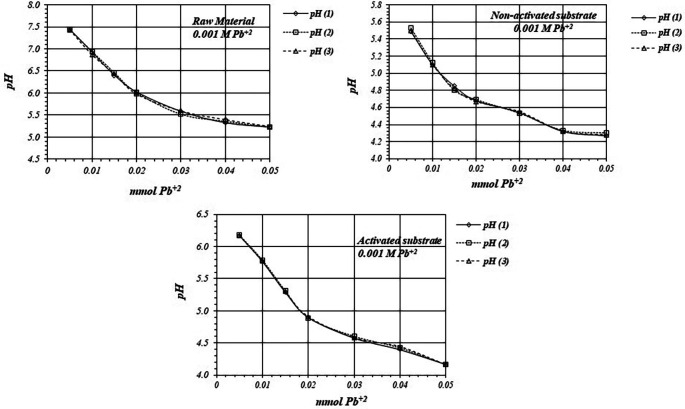
* p*H variation, by triplicate, as a function of mmol of Pb ^+2^ added to 2 *g* of substrate from a 1 *m*M Pb ^+2^ solution, for raw material, and activated and non-activated substrate.

Figure 5 shows *p*H variation during adsorption reaction of Pb ^+2^ ions on raw material, non-activated and activates substrates, by triplicate, from a 1 *m*M Pb ^+2^ ions solution. In all cases adsorption reaction take place with production of protons and solution acidification. Experiments are highly reproducible, so reaction takes place through the same mechanism in all the replicates.

**Figure 6.**
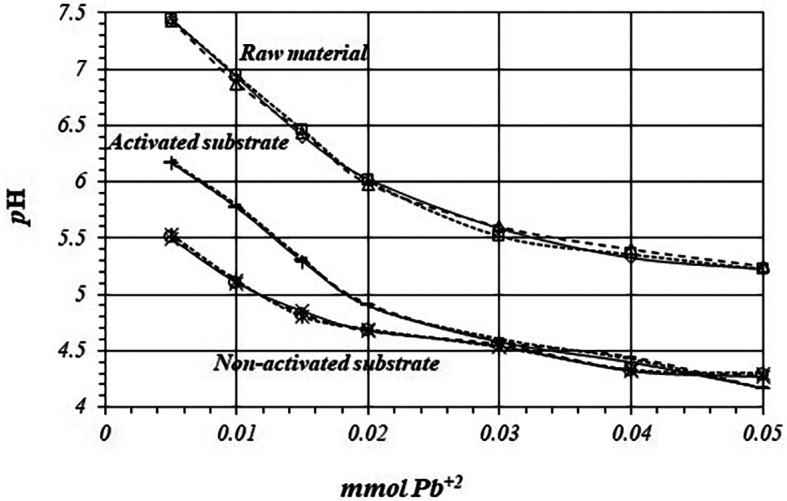
*p*H variation, by triplicate, as a function of mmol of Pb ^+2^ added to 2 *g* of substrate from a 1 *mM* Pb ^+2^ solution, for raw material, activated and non-activated substrate.

Figure 6 shows *p*H variation, by triplicate, as a function of mmol of Pb ^+2^ added to 2 *g* of substrate from a 1 *mM* Pb ^+2^ solution, for raw material, activated and non-activated substrate. On the calcined substrates, the reaction takes place at lower *p*H values, so there are more active sites for the adsorption reaction to occur, with a higher production of protons.

Table 5 shows initial and final concentrations of H ^+^ ions during the adsorption reaction of Pb ^+2^ ions and the net amount of mmol of H ^+^ ions produced in the whole reaction, when a 1 *m*M Pb ^+2^ solution is used. Adsorption reaction on raw material produces 0.29 μmol of H ^+^ and 0.26 μmol on non-activated substrate. However, adsorption reaction on activated substrate produces 0.35 μmol of H ^+^ ion, which is 1.4 times greater than in the case of non-activated substrate.

**Table 5.** mmol of H ^+^ ion produced during the reaction of adsorption when a 1 mM Pb ^+2^ solution is used.

Material	C _i_ H ^+^ mmol/mL	mmol 5 mL	C _f_ H ^+^ mmol/mL	mmol 50 mL	mmol H ^+^ produced
RM	3.63x10 ^-8^	1.82x10 ^-7^	5.89 x 10 ^-6^	2.95 x 10 ^-4^	2.94 x 10 ^-4^
NAS	3.16x10 ^-6^	1.58x10 ^-5^	5.24 x 10 ^-5^	2.62x10 ^-3^	2.60 x 10 ^-3^
AS	6.76x10 ^-7^	3.38x10 ^-6^	6.91 x 10 ^-5^	3.46x10 ^-3^	3.45 x 10 ^-3^

RM: Raw material, NAS: non-activated substrate, AS: activated substrate.

**Figure 7.**
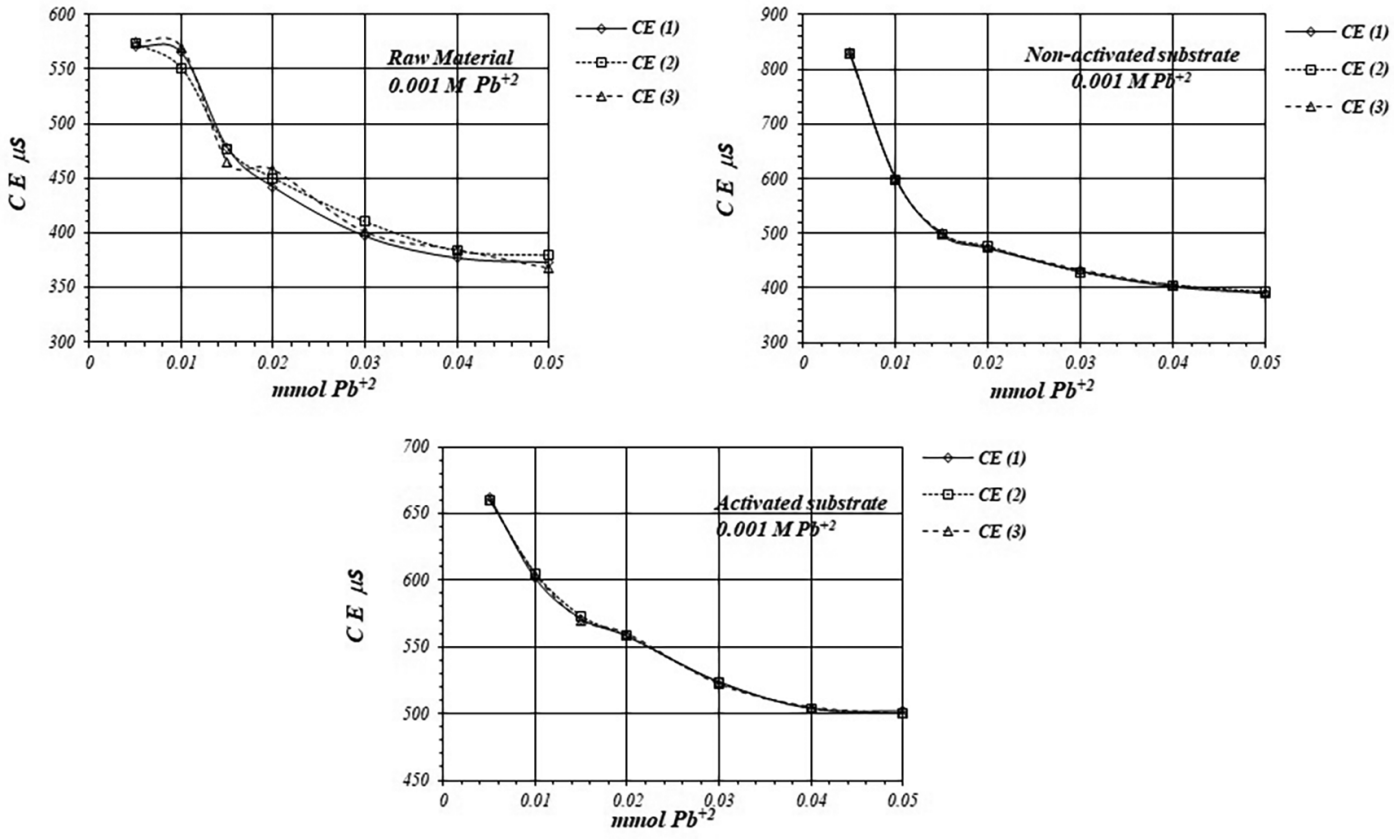
EC variation, by triplicate, as a function of mmol of Pb ^+2^ added to 2 *g* of substrate from a solution 0.001 M Pb ^+2^, for crud material, activated and non-activated substrate.

Figure 7 shows *EC*, variation, by triplicate, during adsorption reaction of Pb ^+2^ ions on raw material and non-activated and activates substrates, when a 1 *m*M Pb ^+2^ solution is used for the reaction of adsorption. *EC* in the solution is basically produced by ions in the solution, *i.e.,* CH _3_COO ^-^ and Pb ^+2^ ions, as well as H ^+^ ions produced in the adsorption reaction. As *EC* decreases, it suggests that Pb ^+2^ ions are adsorbed on the substrates surfaces, as well as on the raw material. The adsorption reaction produces ion immobilization being unable to make a net contribution to the EC of the solution.

**Figure 8.**
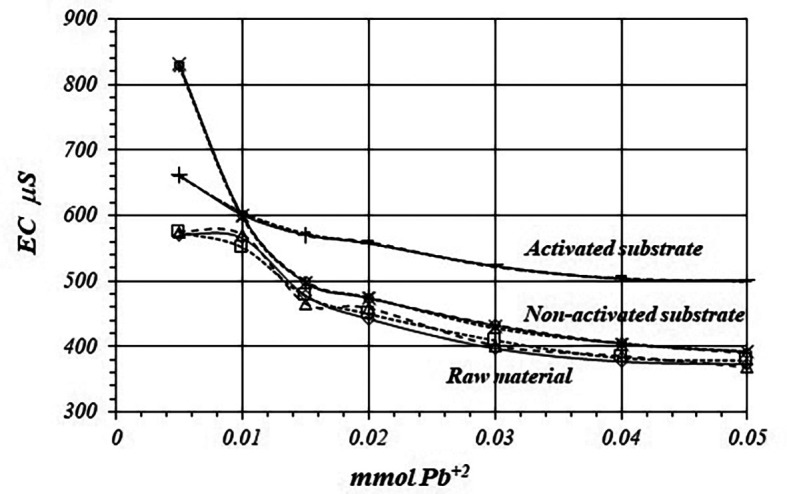
EC variation, by triplicate, as a function of mmol of Pb ^+2^ added to 2 *g* of substrate from a solution 1 *m*M Pb ^+2^, for crud material, activated and non-activated substrate.

Figure 8 shows a comparative view of the *EC* of the solutions for all three cases when a 1 *m*M Pb ^+2^ solution is used. Although conductivity decrease due to adsorption of Pb ^+2^ ions, solution in contact with activated substrate present greater conductivity due to the highest production of H ^+^ ions during the adsorption reaction. These H ^+^ ions have a net contribution to the EC of the solution producing an increasing of the conductivity in the solution which is in contact with the substrates.

**Figure 9.**
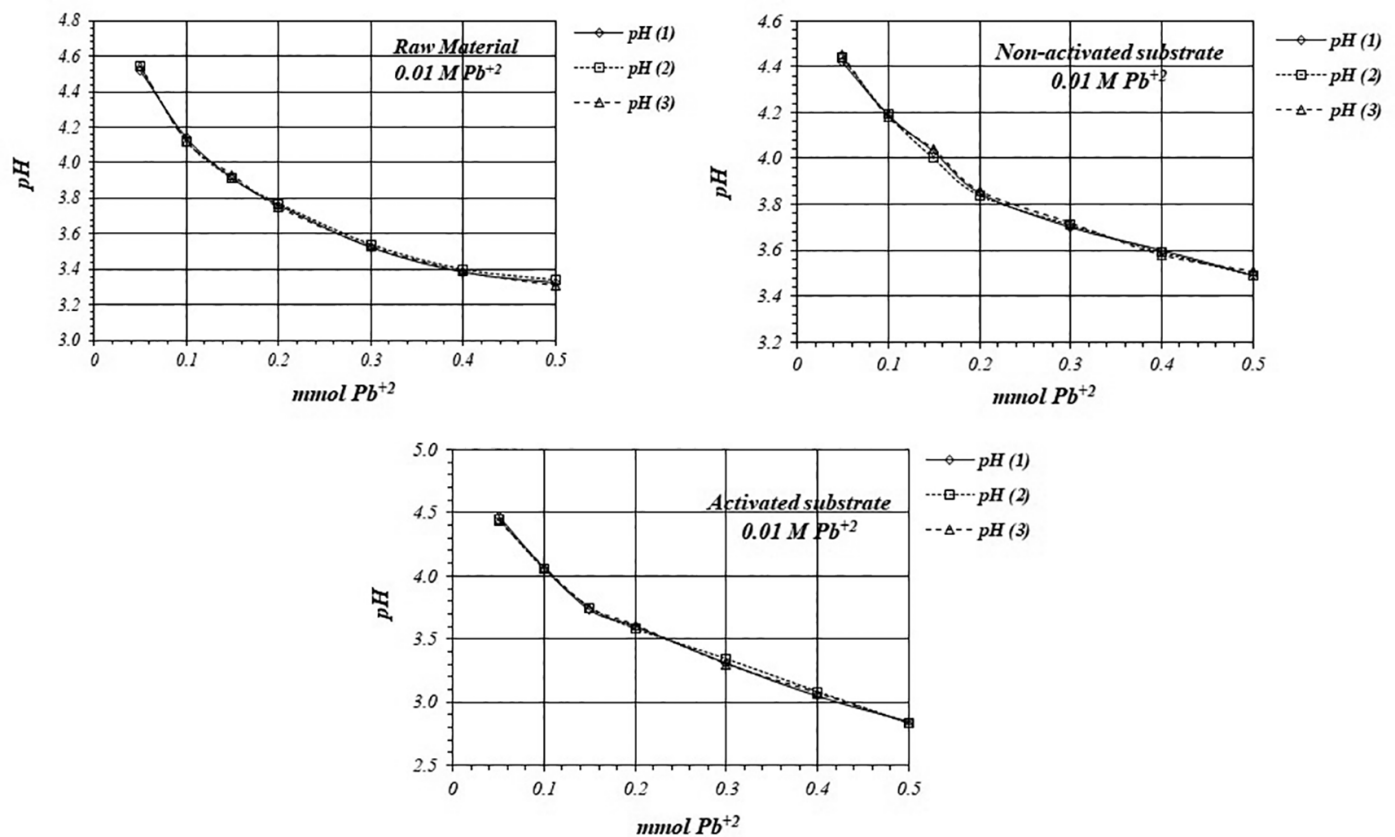
*p*H variation, by triplicate, as a function of mmol of Pb ^+2^ added to 2 g of substrate from a 10 *m*M Pb ^+2^ solution, for raw material, and activated and non-activated substrate.

Figure 9 shows *p*H variation, by triplicate, during adsorption reaction of Pb ^+2^ ions on raw material, non-activated and activates substrates, from a 10 *m*M of Pb ^+2^ ions solution. As in the former cases, the reaction also produces acidification of the solution in contact with the substrates due to the production of H ^+^ ions, which also increases as the concentration of Pb ^+2^ ions increases.

**Figure 10.**
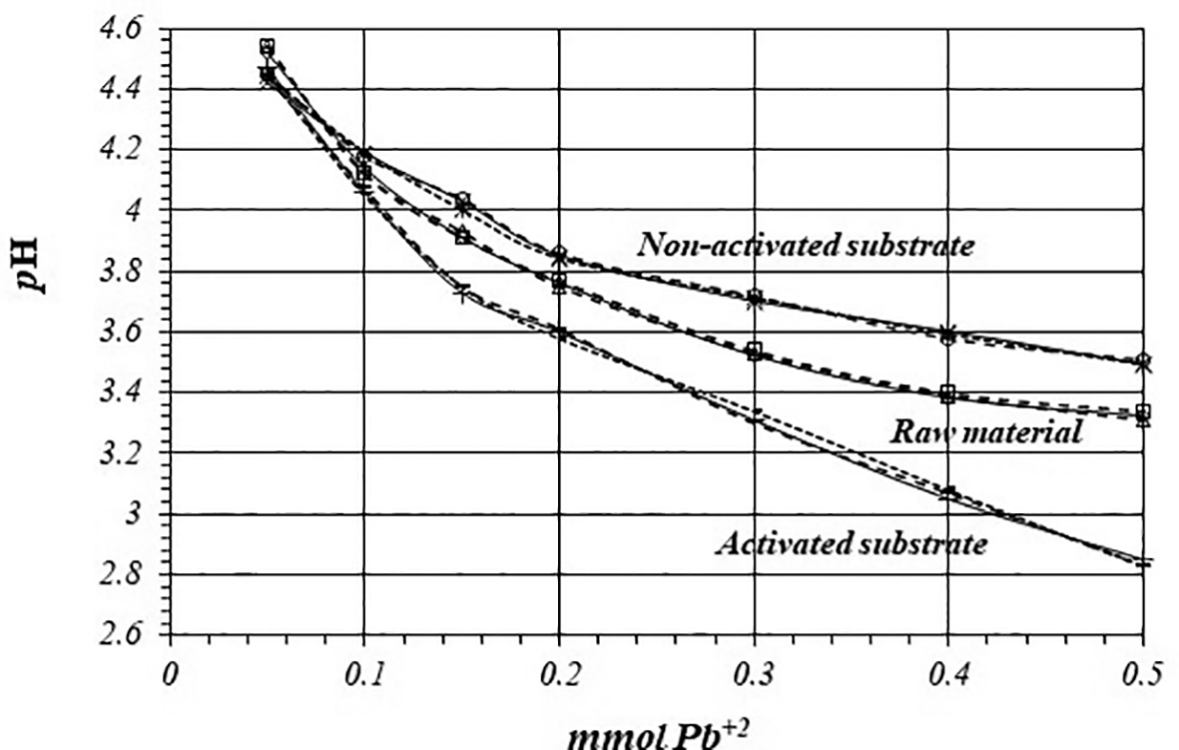
* p*H variation as a function of mmol of Pb ^+2^ added to 2 g of substrate from a solution 10 *m*M Pb ^+2^, for crud material, activated and non-activated substrate.

Figure 10 shows comparative *p*H variations during adsorption reaction on the raw material as well an on the activated ad non-activated substrates when a 10 *m*M of Pb ^+2^ solution is used. As it can be observed, the acidification process is more accentuated in the case of the adsorption reaction on the activated substrate, as is expected. The similarity of the curves is indicative that the experiments are highly reproducible as in the previous case ( Figure 9), so reaction takes place through the same mechanism in all the replicates.

Table 6 shows initial and final concentrations of H ^+^ ions during the adsorption reaction of Pb ^+2^ ions and the net amount of mmol of H ^+^ ions produced in the whole reaction, when a 10 *m*M Pb ^+2^ solution is used. Adsorption reaction on raw material produces 23.3 μmol of H ^+^ ion and 15.6 μmol of H ^+^ on non-activated substrate. However, adsorption reaction on activated substrate produces 72.2 μmol of H ^+^ ion which is 4.6 times greater than in the case of non-activates substrate and 3 times greater than in the case of adsorption reaction on crude material. Moreover, this amount of H ^+^ ions is almost 200 times greater than in the former case when a 1 *m*M Pb ^+2^ solution is used for adsorption reaction.

**Table 6.** mmol of H ^+^ ion produced during the reaction of adsorption when a 10 mM Pb ^+2^ solution is used.

Material	C _i_ H ^+^ mmol/mL	mmol 5 mL	C _f_ H ^+^ mmol/mL	mmol 50 mL	mmol H ^+^ produced
RM	2.95x10 ^-5^	1.47x10 ^-4^	4.68 x 10 ^-4^	0.0234	0.0233
NAS	3.72x10 ^-5^	1.86x10 ^-4^	3.16 x 10 ^-4^	0.0158	0.0156
AS	3.55x10 ^-5^	1.77x10 ^-4^	1.45 x 10 ^-3^	0.0723	0.0722

RM: Raw material, NAS: non-activated substrate, AS: activated substrate.

**Figure 11.**
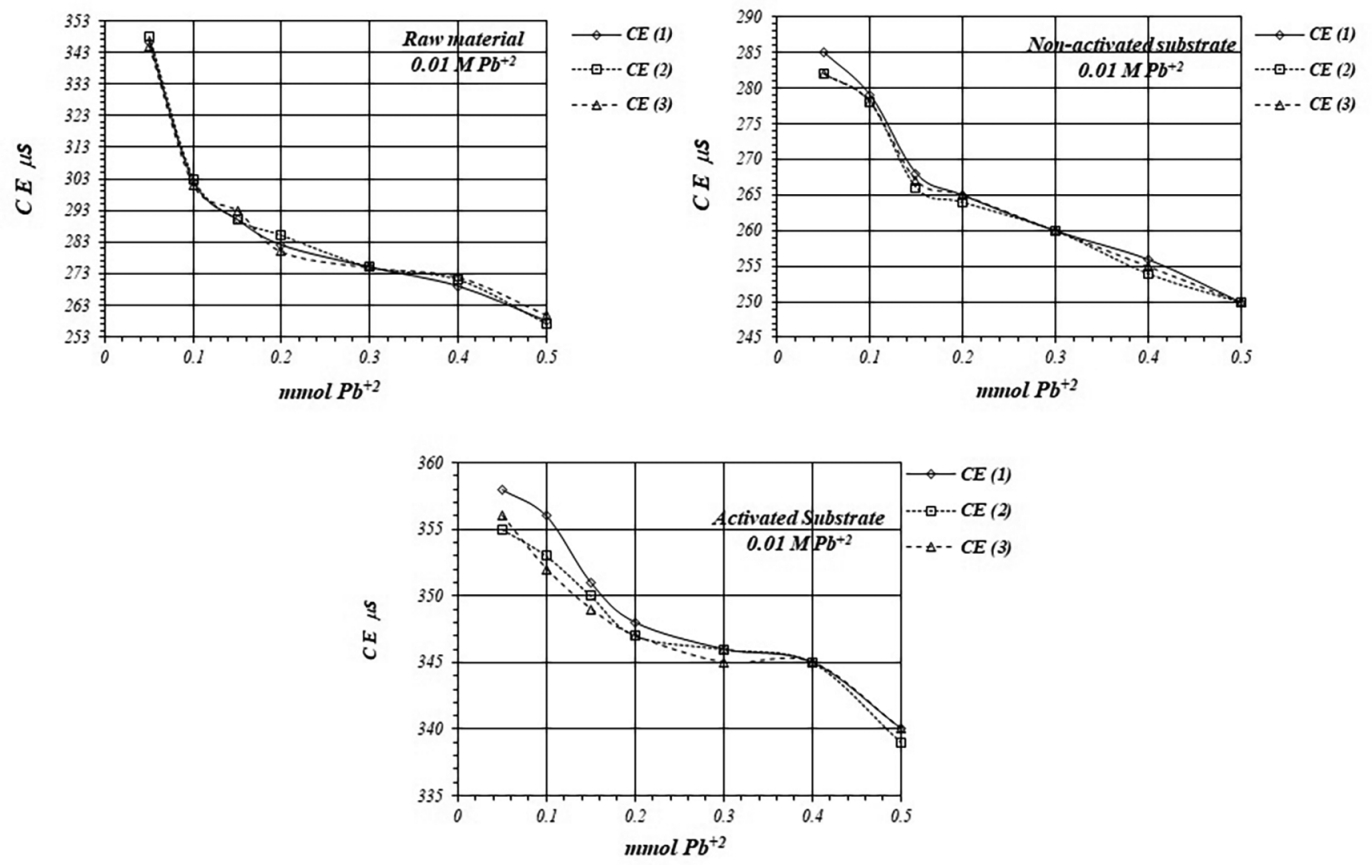
EC variation as a function of mmol of Pb ^+2^ added to 2 g of substrate from a solution 10 *mM* Pb ^+2^, for crud material, activated and non-activated substrate.

**Figure 12.**
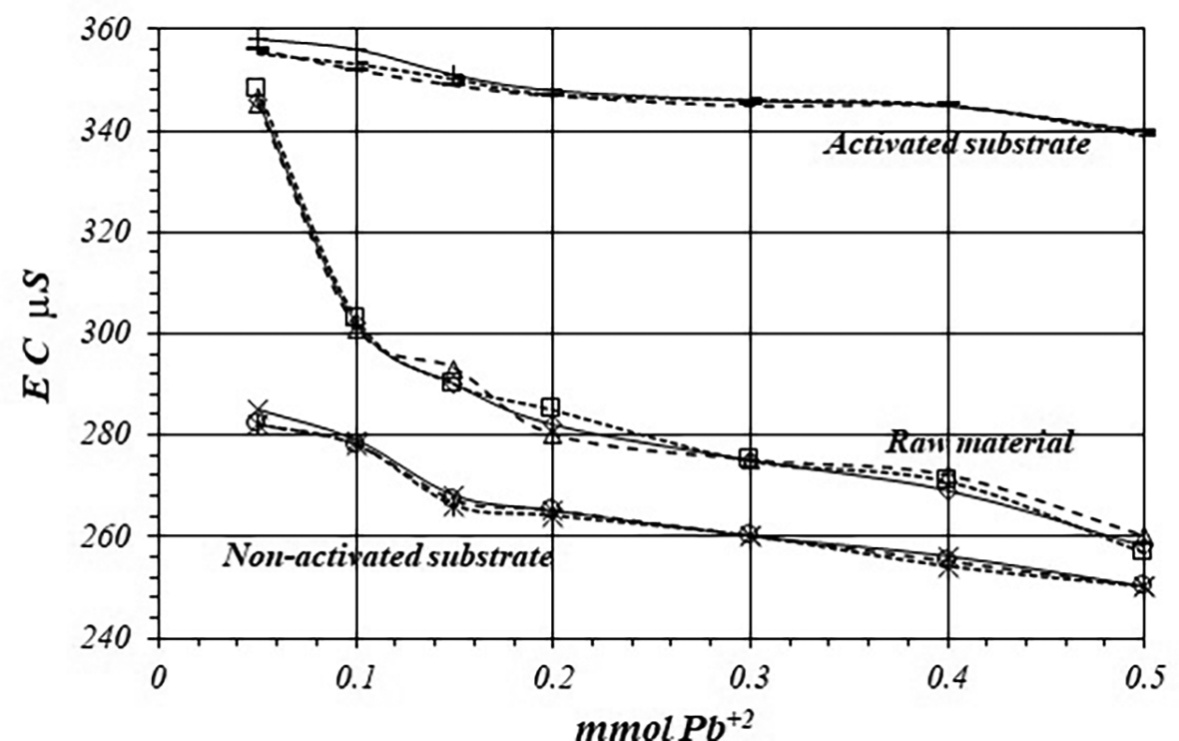
EC variation as a function of mmol of Pb ^+2^ added to 2 g of substrate from a 10 *mM* Pb ^+2^ solution, for raw material, activated and non-activated substrate.

Figures 11 and 12 shows EC, variation by triplicate, during adsorption reaction of Pb ^+2^ ions on raw material and non-activated and activates substrates, when a 10 *m*M Pb ^+2^ is used. As it was pointed out in the former case, EC in the solutions decreases, because Pb ^+2^ ions are adsorbed on the substrate’s surfaces, as well as on the crude material. Nevertheless, the decreasing of the EC in the solutions in contact with the crude material and non-activated substrate is greater due to the minor production of H ^+^ ion during the adsorption reaction, while the EC is greater in the solution in contact with the activated substrate.

## Discussion

The amphoteric oxides of Fe, Al, Mn and Ti presents on the substrate surface, might modify surface charges according to *p*H value, that is to say, in alkaline media, a deprotonation reaction of the oxides take place, increasing surface negative charge density, contrary, in acid media, surface oxide protonation occur, increasing positive charge density according to equation (5) ^35^^–^^37^:

$$H_{2}O+\mathrm{Fe}O^{-}\;\overset{OH^{-}}{\leftarrow }\;\mathrm{Fe}-\mathrm{OH}\;\overset{H^{+}}{\to }\;\mathrm{Fe}-OH_{2}^{+}$$ (5)

Literature ^22^^,^^28^^,^^36^^,^^38^ also suggests a mechanism for the adsorption of transitional metals on these kind of surfaces, through the formation of an inner sphere complex between metal ion and the oxidic surface, according to the equation (6)

$$>\mathrm{Fe}{\mathrm{OH}}^{-1/2}+M{\left(H_{2}O\right)}_{6}^{+n}\to >\mathrm{Fe}-O-M{\left(H_{2}O\right)}_{5}^{+\left(\left(n-3\right)/2\right)}+H_{3}O^{+}$$ (6)

Such a kind of reaction modifies surface charge increasing the positive charge density with the formation of H _3_O ^+^ ion, which could let to anion adsorption producing acidification. This kind of adsorption is defined as specific adsorption or chemisorption, presenting great tendency to irreversibility. ^36^

The isotherm profiles, as it was pointed out before, suggest but don’t confirm information about the interaction between Pb ^+2^ ions and calcined substrate surface, however *L*-type isotherm is indicative of great affinity between Pb ^+2^ ions and calcined substrate surfaces, ^31^^,^^32^^,^^38^^,^^39^ and suggests chemisorption between de Pb ^+2^ ions and the oxidic surface. Literature report similar type of isotherms for other transitional metals as copper in similar kind of substrates ^27^^,^^40^ and variable charge substrate. ^41^^–^^43^ In general, the adsorption phenomenon is most intensive on activates surfaces just because the alkaline solution produces the deprotonation of the oxides, generating negative charges on adsorbent substrate surface according to reaction (5). Therefore, the probability for the Pb ^+2^ ion adsorption is greater on the activated substrate than on the non-activated substrate.

The flat part of the curve ( Figure 1a) suggests formation of a saturated monolayer of Pb ^+2^ ions on the surface as is predicted by the Freundlich and Langmuir models and chemisorption should occur on a single monolayer. This type of isotherm, points to the formation of a covalent bond between Pb ^+2^ ions and the substrate surface that is formed by amphoteric metallic oxides as iron, aluminum, titanium and manganese oxides with variable surface charges. ^19^^,^^26^^,^^37^^,^^43^

However, the Langmuir model has its own limitations when used to explain the adsorption process on non-homogeneous surfaces. This model was developed for gas adsorption on homogeneous surfaces; therefore, the model might fail when adsorption take place on irregular surfaces. One of the disadvantages of this model is to assume the formation of a monolayer on a homogeneous surface where all the available sites for the adsorption are equivalent and the ΔH _ad_ is independent of the degree of surface coverage. However, on a non-homogeneous or irregular surface the adsorption sites are non-equivalent and the ΔH _ad_ varies from one place to another. Consequently, those places which led to a more stable bonding are first occupied. The interaction between adsorbed molecules might affect the affinity between the adsorbate and adsorbent, and as surface coverage increases ΔH _ad_ decreases and also increases the repulsion between the adsorbed molecules. Which at the same time might causes the mobility of the molecules through the surface and different places can be occupied. As a result, a physisorbed layers can be formed over the chemisorbed layer. ^31^^,^^33^^,^^38^^,^^39^

On the contrary Freundlich model adapts better to non-homogeneous surfaces, consequently, the adsorption from aqueous phase on non-homogeneous or irregular surfaces like the calcined substrate surface, the Freundlich model fits better. ^21^^,^^31^^–^^33^^,^^38^

The *p*H measurements during the adsorption reaction showed a significant acidification along the reaction, which agree with the literature ^35^^–^^37^^,^^42^ about transitional metals chemisorption on amphoteric surface with variable charge. This acidification process became more intense as the concentration of Pb ^+2^ ions in the solution increases. These facts, the type of adsorption suggested by the isotherm and the acidification process, could be interpreted in terms of a covalence formation between Pb ^+2^ ions and the calcined substrate in a specific adsorption reaction or chemisorption, according to the model presented in the equation 7 suggest by the literature. ^19^^,^^27^^,^^35^

$$>M-{\mathrm{OH}}^{-0.5}+\mathrm{Pb}{\left(H_{2}O\right)}_{4}^{+2}\to \left[\left(>M-O\right)\right]-\mathrm{Pb}{\left(H_{2}O\right)}_{3}^{+0.5}+H_{3}O^{+}\;$$ (7)

According to the results in this investigation, the adsorption reaction is more favorable on the activated substrate where more adsorption active sites are available. The small negative charge density on the non-activated substrate is a limiting factor for the adsorption to occur. Similar results have been reported in the literature for the adsorption of Cu ^+2^ ions. ^27^^,^^40^^,^^43^ Therefore, it is expected that other transitional metals can suffer such a kind of reaction on the surface of these kinds of substrates, being possible their separation from contaminated waters during a filtration process in a granular media.

In general, adjustment data by Freundlich and Langmuir models fits well with experimental data, especially for activated adsorbent substrates. However, in both cases calculated values are biased from the experimental data, especially for the Langmuir model, probably because on the irregular and roughness surface. This represent less homogeneity of the negative charge density on the surface, so not all adsorbent positions are equivalent. Therefore, the condition that surfaces must have positions for adsorption, which are all equivalents and only one molecule or ion can occupy one single place is no longer fulfilled. In fact, both models pursue to explain the same type of isotherm but Freundlich is an empirical model with less straightening conditions compared to the Langmuir which was developed on valuable ideal theoretical considerations difficult to respect in the case of the calcined substrate.

### Ethical considerations

Not applicable.

## Data Availability

Figshare: Adsorption of Pb (II) ions on Variable Charge Oxidic Calcined Substrates with Chemically Modified Surface https://doi.org/10.6084/m9.figshare.22266772.v2. ^34^ This project contains the following underlying data:
- Study Adsorption Pb Freundlich model.xlsx - Study Adsorption Pb Langmuir model.xlsx - Study Adsorption Pb H+ produced.xlsx - Study pH Adsorption Pb.xlsx - Study CE Adsorption Pb.xlsx
