# Adsorption of Pb (II) ions on variable charge oxidic calcined substrates with chemically modified surface

**DOI:** 10.12688/f1000research.132880.2

**Published:** 2024-03-28

**Authors:** José G. Prato, Fernando Millán, Marialy Rangel, Andrés Márquez, Luisa Carolina González, Iván Ríos, César García, Carlos Rondón, Enju Wang

**Affiliations:** 1 Escuela de Ingeniería Química, Facultad de Ingeniería, Universidad de Los Andes, Mérida, 5101, Venezuela; 2 Grupo de Investigación Estudios Interdisciplinarios, Ingeniería Ambiental, Facultad de Ingeniería, Universidad Nacional de Chimborazo, Riobamba, Chimborazo Province, 060103, Ecuador; 3 Chemical Engineering School, Polytechnical Institute Santiago Mariño, IUPSM-Mérida, Mérida, 5101, Venezuela; 4 Facultad de Farmacia y Bioanálisis, Universidad de Los Andes, Mérida, 5101, Venezuela; 5 Grupo de Investigación “Análisis de Muestras Biológicas y Forenses”, Laboratorio Clínico, Facultad de Ciencias de la Salud, Universidad Nacional de Chimborazo, Riobamba, Chimborazo Province, 060103, Ecuador; 6 Arquitectura, Facultad de Ingeniería, Universidad Nacional de Chimborazo, Riobamba, Chimborazo Province, 060103, Ecuador; 7 Departamento de Química, Facultad de Ciencias, Universidad de Los Andes, Mérida, 5101, Venezuela; 8 Department of Chemistry, Saint John´s University, Jamaica, NY, 11439, USA

**Keywords:** ionic adsorption, calcined substrate, Pb(II) ions, isotherms

## Abstract

**Background:**

The paper describes lead ion adsorption on variable charge oxidic calcined substrates with chemically modified surfaces. Amphoteric oxides of iron, aluminum, titanium, and manganese, change their surface electric charge after acid or alkaline treatment, letting cationic or anionic adsorption reactions from aqueous solutions. This property allows using them as adsorbing substrate for heavy metals retention in water treatment systems.

**Methods:**

Substrate was prepared by extruding cylindrical strips from a saturate paste of the oxidic lithological material-OLM; dries it up and thermally treated by calcination. The study was performed by triplicated trial, on batch mode, using 2 grams samples of treated with NaOH 0.1N and non-treated substrate. Lead analysis was performed by AAS-GF. Freundlich and Langmuir models were used to fit results. Comparing differential behavior between treated and non-treated substrates showed the variable charge nature of the OLM.

**Results:**

Results show *L*-type isotherms for the adsorption of Pb(II) ions on the activated substrate, suggesting good affinity between Pb(II) ions and OLM’s surface. Average value of adsorption capacity ( *K*) for activated substrate (1791.73±13.06), is around four times greater than the non-activated substrate (491.54±31.97), during the adsorption reaction, 0.35 and 0.26 mmolH ^+^ of proton are produced on the activated and non-activated substrate respectively using a 1 *m*M Pb(II) solution and 72.2 and 15.6 mmolH ^+^ using a 10 *m*M Pb(II) solution. This acidification agrees with the theoretic model of transitional metals chemisorption on amphoteric oxides, present in lithological material used for the preparation of adsorbent substrates, confirming the information given by the *L*-type isotherms.

**Conclusions:**

Results suggest that these variable charge oxidic adsorbent substrate show great potential as an alternative technique for water treatment at small and medium scale using granular filtration system. The easiness and low price make them suitable to apply in rural media where no treating water systems is available.

## Introduction

“ *Lithologic materials*” are all those materials belonging to the Earth crust that cannot be classified as soil. However, some of these materials behave as arid soils basically composed by refractory aluminum and iron oxides among other crystalline phases as quartz and clays, they are called “Oxidic Lithologic Material”, OLM. ^1, 2^ The two most important properties of these kinds of materials are the thermal resistance and their amphoteric behavior. ^2, 3^ In alkaline medium the oxides deprotonate, to create surface negative charges while in acidic medium the oxides protonate, to create surface positive charges. In the first case it favors the cationic adsorption while the second one it favors the anionic adsorption. ^2, 4– 7^ Their thermal resistance allows the preparation of an adsorbent granular medium for water treatment at low cost. As an example of the usefulness of such kind of calcined oxidic substrate could be the removing of heavy metals from water.

Lead (Pb) is one of the top ten toxic substances with a great distribution in the environment, ^8, 9^ it reaches water sources through various anthropogenic activities such as leaded pipe corrosion or non-treated industrial wastes, as ceramics factories industrial mining activities, oil refining, fossil fuels combustion, frosted lead paints, accumulators manufacturing, among other industrial activities, ^9, 10^ leaded fuels are still used in many South America countries, with great environmental impact. ^11^ Lead pipes have been used for water supply network until the seventies however, old constructions still remain and new residential buildings contain plumbing devices for water services. ^12, 13^ Recently lead-contaminated water in some High Schools in Brooklyn, New York has been detected at high concentrations, exceeding the Environmental Protection Agency’s (EPA’s) action level of 0.015 mg/L. ^12^ Depending on the local water characteristics, such as *p*H, hardness and temperature, the lead in the pipes and faucets can dissolve, becoming a health risk for the consumers. ^13^ Lead intake in human being can take place basically via ingests, food or water or breathing, dust in the air might serve as a transport media for lead to travel. ^9, 12^ Lead is especially harmful for kids, it accumulates in the soft tissues and bonds, being difficult to eliminate. After accumulation, lead attacks the brain and the central nervous system, with permanents sequels. One of these consequences is the child development delay and cognitive disability. ^13^

The conventional methods for removing heavy metals from water and sewage have been well described in literature. There are a great variety of methods including chemical precipitation, ^8– 10^ ion exchange, ^8, 9^ reverse osmosis, ^10^ membrane processes, ^14^ photocatalysis, ^15^ microbial biotechnology, ^8, 9^ coagulation, ^16– 18^ flocculation, ^10^ filtration ^17^ and adsorption technology. ^14– 17^ Some of them, such as precipitation, coagulation and sedimentation have disadvantages of generating sludge that requires further treatment, which increases operational costs. ^14, 16– 18^ Ion exchange, reverse osmosis, membrane processes are expensive, need technical assistance for maintenance and few supply companies can offer such kind of systems. ^8– 10^

Of all these methods, adsorption technology is one of the most commonly used treatment method due to its advantages: ease to operate, regeneration potentials, lack of sludge formation, inertness to materials and relative low cost. ^9, 10, 18, 19^ A great variety of adsorbents have been tried and studied, different types of biomass and organic adsorbents as agricultural by-products and its conversion to activated carbon, ^16– 18, 20^ biopolymers, ^17, 21^ fungal biomasses have been used with 80 to 100 % percentage retention. ^22^ Rice straw derived biochar have been used as amendment in soils for Pb immobilization, avoiding its run off through the soil and protect the rivers. ^23^ The use of Nanomaterials for adsorption processes have been widely reported, their physicochemical properties as small size and large surface area, make them suitable for water treatment systems. Amorphous nanoaluminophosphates have been used for lead retention from aqueous solution with yield between 40 and 70%, ^24^ diverse carbon nanotubes technologies have been tried for the adsorption of various hazardous metals as As, Cd, Pb, Cr, Ni, Cu, Zn, with adsorption capacities up to 500 mg Pb(II)/g. ^10, 14, 18^ However, most of these methods, are expensive, less available or difficult application at medium and large scale.

In previous studies some oxidic lithologic materials, OLM have been characterized with the purpose of preparing calcined adsorbent substrates for ionic adsorption from aqueous solution. ^1, 2, 25, 26^ The high content of amphoteric iron and aluminium, as well as titanium and manganese oxides, with *p*H dependent variable charges surfaces, make them suitable for ionic adsorption. ^2, 5, 7^ As a consequence of this particular property, these OLM materials are versatile for preparing a calcined adsorbing substrate using thermal treatment of calcination. Positive or negative charges density is achieved by alkaline or acid treatment, alkaline treatment causes oxide deprotonation creating a negative charge density while acid attack causes oxide protonation, creating a positive charge density on the oxide surface. Such kind of substrate has been applied in water softening, ^25, 27, 28^ suggesting that alkaline and alkaline-earth metals participate in cationic exchange reactions. Other studies suggest that transitional metals as copper, zinc and chrome participate in chemisorption reactions. ^1, 29, 30^ These studies suggest that other transitional metals may also participate in similar kind of specific adsorption reaction. ^2, 6^ Residual water treatment and organic matter removing also have been studied. ^3, 31^ This study reports a 60-85% reduction in turbidity units, 95-98% reducing biological oxygen demand as well as 88-94% reducing chemical oxygen demand. After acid treatment, the calcined substrate has also been applied in anionic adsorption studies as sulphate and phosphate adsorption. ^2, 32, 33^ All these studies have confirmed that adsorption reaction is more efficient and better defined on the treated substrate than on non-treated substrate. The chemical treatment modifies the surface charge and creates a more homogenous distribution of sites suitable for adsorption. Based on the previous findings, the main objective of the present study is to apply this new kind of substrate in the study of the relative affinity between the Pb(II) ions and the oxidic calcined surface, in order to use it as a granular medium for water treatment.

## Methods

### Reagents

All the reagents used in the experimental phase are analytical grade, Merck reagent: (CH _3_COO) _2_Pb, NaOH, HCl and distillate water.

### Oxidic lithological material

A 5 kg sample of the raw OLM was collected from a natural deposit, located at the coodinates 8°28′47″ N and 71°23′47″ W. This is an arid zone, where the temperature varies fom 17 to 30 °C, along the year and maximum rainfall of 200 mm per year. The OLM have been previously characterized and the results are reported in the literature. ^1, 2, 25, 26, 28^ A brick-red colored sandy loam material with relative low exchange capacity and very low organic material content. Important metallic content: Al (11.75 %), Fe (7.24 %), Ti (0.37%) and Mn (0.03%) are the major metals, ^1^ these metals are present as refratory amphoteric oxides. Alkaline and alkaline earth content between 0.01 and 1%, with Na (0.86%) and K (1.62%) and very few Ca (0.032%) and Mg (0.31%) content. ^26^ Several transitoal metals as traces. Due to its thermal resistence, this OLM is used by potters for preparing bricks by thermal treatment.

### Preparation of the adsorbent substrate and activation of negative surface charges

The OLM was crushed using a rubber hammer to avoid the destruction of the mineral structures, sieved for 5 min, using an Octagon 200CL Digital Sieve Shaker (Endecotts Ltd, England) to obtain particle-size fractions of 800 μm, then mixed with distillated water in order to obtain a homogeneous saturated paste easily moldable. 3 mm diameter cylindrical strips were extruded with the a 60 mL syringe, cut into 5 mm long pieces in order to prepare a granular medium (Average diameter: 3.52 ± 0.28 mm, average length: 5.43 ± 0.66 mm, pellet average volume: 51.29 ± 1.70 mm ^3^). The drying process takes place in two steps, for the first step, the granular substrate is air dried for 24 hours, in the second one the substrate is oven drying during another 24 hours, using an oven FELISA FE-293, Jalisco, Mexico, at 150 °C. These two drying steps assure the total elimination of water from the substrate. If there is occluded water it will explode during the calcination process, and might destroy the substrate pellets. Finally, the dried solid substrate is calcined up to 750 °C for four hours in a Thermolyne FB141OM furnace, Thermo Scientific, Waltham, USA, and then cooled down for 12 hours, until 20 °C, before opening the furnace door. The calcination process favors the oxides formation, at such a high temperature, oxygen is very reactive, reacting with the metal to form oxides. Also favor the cementation of the pellets, avoiding dispersion in the aqueous solution. Finally, during the calcination process, organic matter fraction, which is very small, is complete burned out, so only the oxidic phase participates in the adsorption reaction. However, during calcination process the specific surface might be reduced.

The calcined substrate is chemically treated in alkaline media with 0.1 N NaOH solution for 12 hours in a flask at room temperature. The alcaline causes the deprotnation of the oxides, enhancing the negative charge density on the surface, allowing the cationic adsorption. The chemically treated substrate is labeled as activated substrate and the non-treated substrate is labeled as non-activated substrate. After chemical treatment, the substrate is washed out with distilled water until neutral *p*H, then oven dried at 120 °C for 12 hours. The calcined substrate is saturated with distilated water before adsorption, to asure the ionic mobility and reach equiilibium faster.

### Adsorption studies

The adsorption study was performed in triplicated trial, in isothermal conditions (20 ± 2 °C) using batch equilibration procedure, treating seven samples of 2 g of activated and non-activated calcined substrate, with increasing aliquots of 5, 10, 15, 20, 25, 30 and 40 mL from the 1 *m*M Pb(II) solution, for 24 h. Suspensions were periodically shaken every hour. Differences between *C*_*i*_ and *C*_eq_ were assumed to be due to adsorption. Adsorption isotherms were obtained by plotting the amount of lead adsorbed ( *q*_*e*_), against the Pb(II) equilibrium concentration ( *C*_eq_) and fitted to the linear forms of the Freundlich and Langmuir equations. ^2, 9, 18, 25, 34– 37^ Comparison between activated and non actvated substrate will show evidence that the oxide deprotonation reaction by the alkaline treatment on the activated substrate.

Freundlich Isotherm is an empirical model which assumes an adsorption process characterized by mulitilayer adsorption on heterogeneous surfaces. The model is described by the equation 1 and the linear form by equation 2. ^2, 9, 34, 35, 38^ A graph representation of log ( *q*_*e*_) *vs* log ( *C*_eq_) should be a straight line, with slope equal to 1/ *n* and intercept equal to log ( *K*_*F*_):

$$q_{e}=K_{F}*C_{\mathrm{eq}}^{\frac{1}{n}}$$ (1)

$$\log\left(q_{e}\right)=\log\left(K_{F}\right)+\frac{1}{n}\;\log\left(C_{\mathrm{eq}}\right)$$ (2)

where *q _e_* is the amount adsorbed per adsorbent weight unit, *C*_eq_ is the equilibrium concentration of adsorbate in solution after adsorption. *K*_*F*_ is the Freundlich constant related to adsorption capacity and *n* is a constant related to adsorption intensity or energetic homogeneity of active sites of adsorption. *n* may take values near unity or greater. The lower the value of *n* is, the lower the energy heterogeneity in the active adsorption sites.

The Langmuir model describes a reversible process with the formation of adsorbate monolayers on the adsorbent surface. The nonlinear and linear forms of the Langmuir isotherm are described in Equations (3) and (4) ^9, 10, 18, 25, 37^:

$$q_{e}=\frac{k_{1}*k_{2}*C_{\mathrm{eq}}}{\left(1+k_{1}*C_{\mathrm{eq}}\right)}$$ (3)

$$\frac{C_{\mathrm{eq}}}{q_{e}}=\frac{1}{k_{1}*k_{2}}+\frac{C_{\mathrm{eq}}}{k_{2}}$$ (4)

where *k*_1_ is the Langmuir constant related to the affinity between adsorbate and adsorbent and *k*_2_ is the adsorption capacity, which represents the maximum amount of adsorbate in a monolayer. A graph representation of *C*_eq_/ *q*_*e*_ *vs* *C*_eq_ should be a straight line, with slope equal to 1/ *k*_2_ and intercept equal to 1/( *k*_1_* *k*_2_). *k*_2_ and *k*_1_ are determined by the straight line from the slope and the intercept respectively. Values of *k*_1_ and *k*_2_ are then introduced in equation (4) to determine the calculated value of calculated *q*_*e*_ and then compared with experimental value.

The best fit between the isotherm function and the experimental data was verified through linear regressions of the isotherm linear equation. The fitting of the isotherms was verified through the comparison of the experimental ( *q*_*e*_ exp) and calculated ( *q*_*e*_ calc) value obtained from the isotherm equation, by means of the linear correlation between the two values, given by the linear regression coefficient ( *r*).

### Lead analysis

Pb(II) analyses were performed by AASGF, using a Varian Graphite Furnace Atomic Absorption Spectrophotometer, Spectra AA Zeeman 220 (Palo Alto, California, USA), with a pyrolytic coated graphite tube and Zeeman background corrector. The spectrophotometer is coupled to a Varian auto sampler model EL-97113008. A Varian Uranium hollow cathode lamp was used and measured at 283.3 nm.

### *p*H and electric conductivity studies

Solution *p*H and electric conductivity (EC) were measured by triplicate trial, using the same isothermal batch equilibration. *p*H was measured with a HI 211 *p*Hmeter (HANNA instruments, Smithfield, Rhode Island, USA), calibrated with commercial buffer solutions of *p*H 4 and 7. EC was measured with a Trans Instrument HC3010 Conductimeter (Petro Centre, Bukit Merah, Singapure), calibrated with standard reference. All experimental data ^39^ was managed with the Excel software (Microsoft, Los Angeles, USA).

## Results

### Isotherm graph

**Figure 1.**
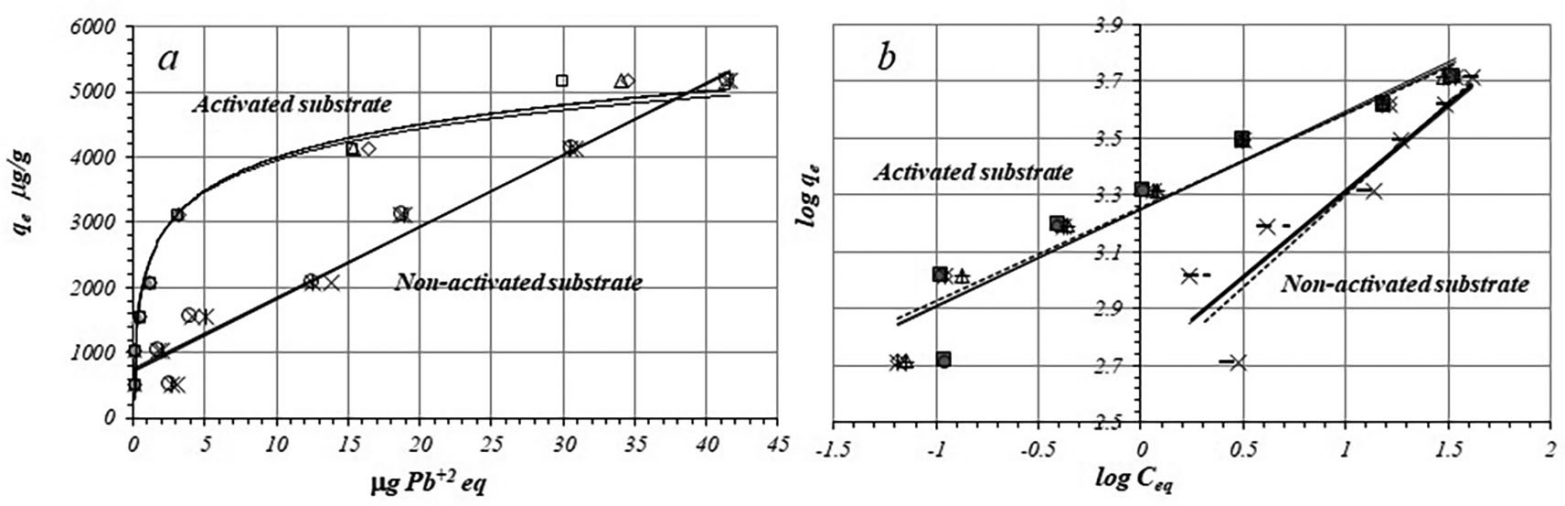
*a*: Isotherms for Pb(II) ions adsorption and *b*: fitting to the logarithmic form of the Freundlich model for the activated and non-activated substrates.

Figure 1a shows by triplicated trial, the adsorption isotherms of Pb(II) ions on the activated and non-activated substrates. Lead adsorption on the activated substrate follow an *L*-Type isotherm model, while the adsorption on the non-activated substrate looks more like a linear model.

Figure 1b shows linear fitting of adsorption data to the Freundlich model. Table 1 shows the fitted equations according to the Freundlich model, as well as parameters *r*, *K _F_*, and *n* values for activated and non-activated substrates, by triplicated trials. *r* values look more favorable for the activated substrate, *K* *_F_* value (1791.73 ± 13.06 RSD 0.72%) is about 4 times greater than the respective *K* *_F_* value for the non-activated substrate (491.54 ± 31.97 RSD 6.5%). Similarly, the *n* values suggest greater adsorption intensity on the activated substrate in relation to the non-activate substrate.

**Table 1.** Freundlich model parameters, *r*, *K*_F_, and *n* values for activated and non-activated substrates, by triplicated.

Substrate	Fitted equation	*r*	*K*_F_	*n*
Activated	y=3.2523+0.3446x	0.9733	1787.72	2.9
	y=3.2568+0.3294x	0.9713	1806.34	3.0
	y=3.2507+0.3366x	0.9497	1781.15	3.0
Non-activated	y=2.7061+0.5998x	0.9098	508.28	1.7
	y=2.6577+0.6434x	0.9423	454.67	1.6
	y=2.7090+0.6052x	0.9268	511.68	1.7

**Figure 2.**
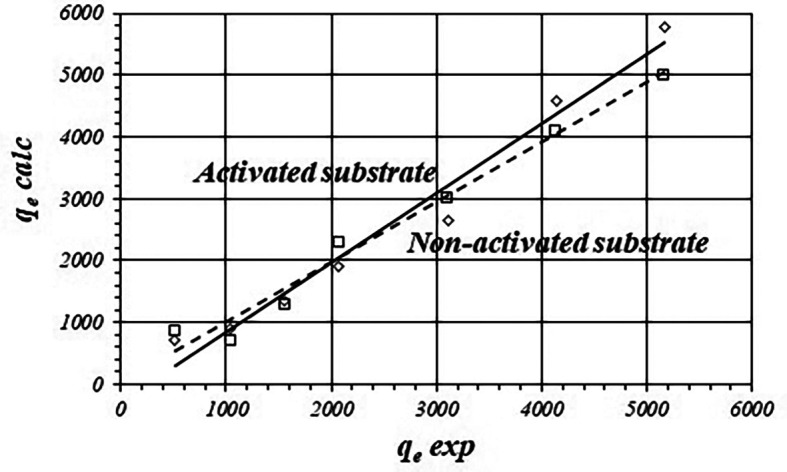
Correlations between calculated and experimental values of *q _e_* (Freundlich model) for the activated and non-activated substrates.

Figure 2 shows the correlation between calculated and experimental *q _e_* data for the activated and non-activated substrates and Table 2 shows fitted equations for the linear functions. For the activated substrate, calculated values of *q _e_* are biased from the experimental data about 15.16 ± 6.63% and 17.41 ± 18.15% for the non-activated substrate.

**Table 2.** Fitted equations for the correlation between *q _e_* calculated and *q _e_* experimental (Freundlich model).

Substrate	Fitted equation	*r*
Activated	y=−270.63+1.1222x	0.9858
Non-activated	y=44.206+0.9671x	0.9893

**Figure 3.**
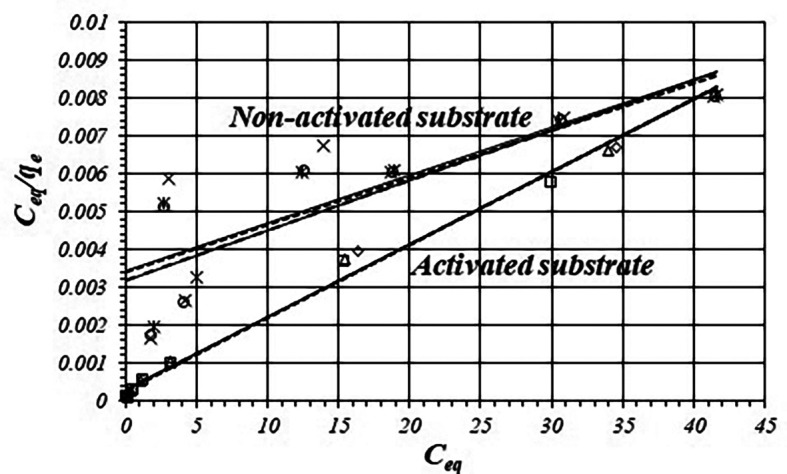
Fitting isotherms to the linear form of the Langmuir equation for the activated and non-activated substrates.

Figure 3 shows fitting of adsorption data according to the Langmuir model and Table 3 shows fitted equations, *r*, *k*_1_ and *k*_2_ values for the activated and non-activated substrates, by triplicated. *r* values are more favorable for the activated substrate than in the non-activated substrate. The *k*_1_ value for the activated substrate is about 20 times greater than for the non-activated substrate.

**Table 3.** Langmuir model parameters, *r*, *k*_1_ and *k*_2_ values for activated and non-activated substrates, by triplicated.

Substrate	Fitted equation	*r*	*k*_1_	*k*_2_
Activated	y=0.0003+0.0002x	0.9937	0.6667	5000
	y=0.0003+0.0002x	0.9945	0.6667	5000
	y=0.0003+0.0002x	0.9950	0.6667	5000
Non-activated	y=0.0034+0.0001x	0.7993	0.0294	10000
	y=0.0034+0.0001x	0.8638	0.0294	10000
	y=0.0032+0.0001x	0.8578	0.0294	10000

**Figure 4.**
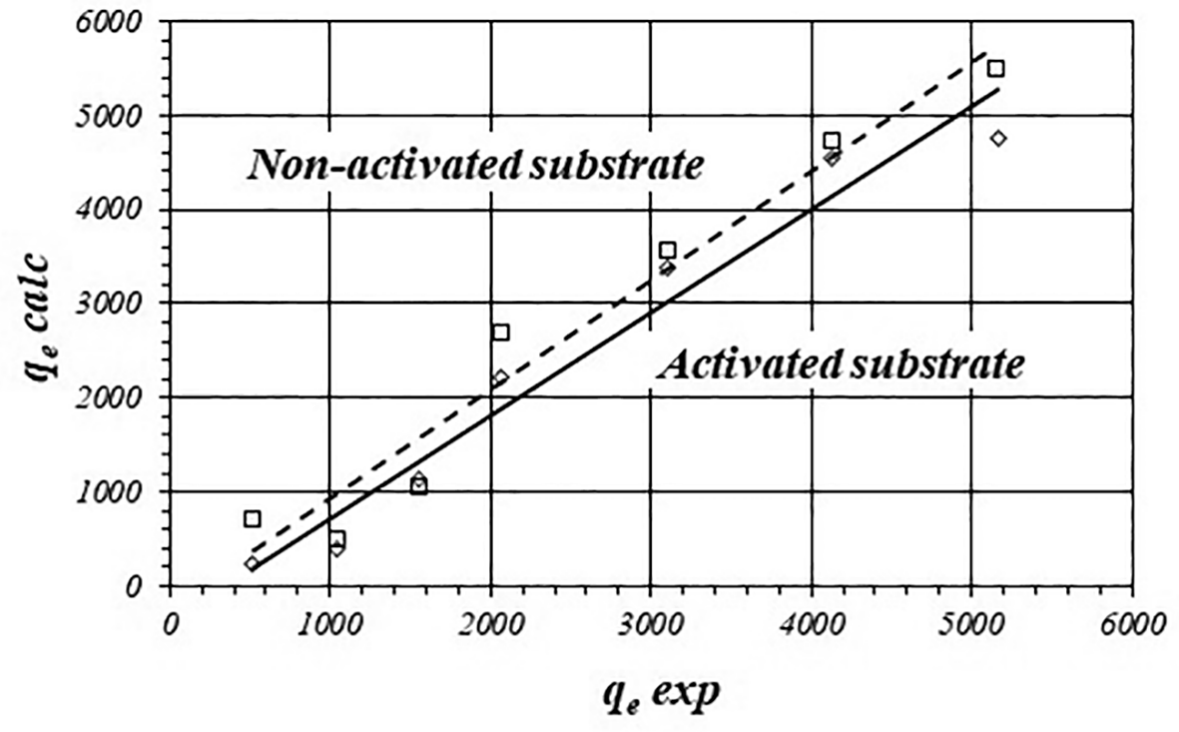
Correlations between calculated value of *C*_ad_ *vs* experimental value (Langmuir model) for the activated and non-activated substrates.

Figure 4 show the correlations between calculated and experimental values of *q*_e_ for the activated and non-activated substrates and Table 4 shows fitted equations for the linear functions. Calculated values of *q*_e_ are even more biased from the experimental data compared with the Freundlich model. For the activated substrate the average difference is about 25.29 ± 23.67% and 27.22 ± 16.02% for the non-activated substrate.

**Table 4.** Correlations between calculated and experimental value of *q _e_* (Langmuir model) for the activated and non-activated substrates.

Substrate	Fitted equation	*r*
Activated	y=−365.81+1.0936x	0.9809
Non-activated	y=−221.28+1.1581x	0.9784

### *p*H and electric conductivity study

**Figure 5.**
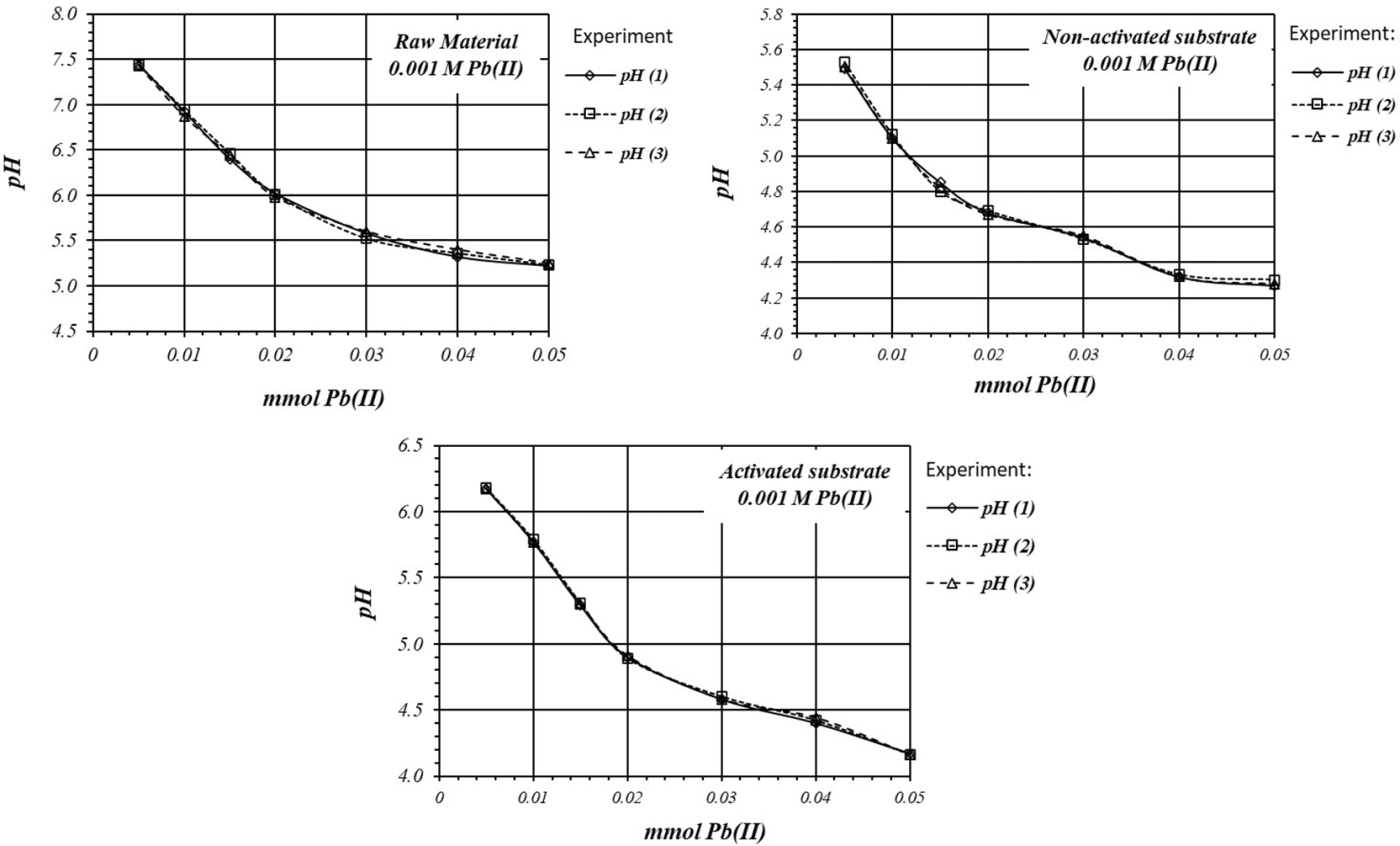
*p*H variation, by triplicate, as a function of mmol of Pb(II) added to 2 *g* of substrate from a 1 *m*M Pb(II) solution, for raw material, and activated and non-activated substrate.

Figure 5 shows by triplicated trial, *p*H variation during adsorption reaction, as a function of mmol of Pb(II) added to the activated, non-activated substrate and raw material, using a 1 *m*M Pb(II) ions solution. In all cases adsorption reaction take place with solution acidification. Experiments are highly reproducible, so reaction follows the same mechanism in all the replicates.

**Figure 6.**
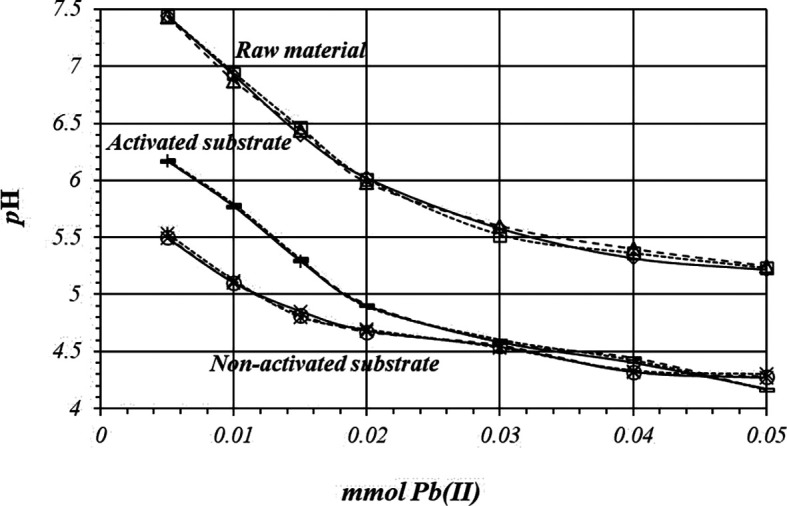
*p*H variation, by triplicate, as a function of mmol of Pb(II) added to 2 *g* of substrate from a 1 *m*M Pb(II) solution, for raw material, activated and non-activated substrate.

Figure 6 shows by triplicate, the relative comparison of *p*H variation, for all these three cases. The acidification is higher on the activated substrates than in the non-activated substrate or in the raw material, so there are more active sites for the adsorption reaction to occur, with a higher production of protons.

Table 5 shows initial and final concentrations of H ^+^ ions during the adsorption reaction of Pb(II) ions and the net amount of mmol of H ^+^ ions produced in the whole reaction, when a 1 *m*M Pb(II) solution is used. Adsorption reaction on the activated substrate produces 0.35 μmol of H ^+^ ion, compared with the 0.26 μmol of H ^+^ on the non-activated substrate, while raw material produces 0.29 μmol of H ^+^.

**Table 5.** mmol of H ^+^ ion produced during the reaction of adsorption when a 1 *m*M Pb(II) solution is used.

Material	C _i_ H ^+^ mmol/mL	mmol 5 mL	C _f_ H ^+^ mmol/mL	mmol 50 mL	mmol H ^+^ produced
RM	3.63 x 10 ^-8^	1.82 x 10 ^-7^	5.89 x 10 ^-6^	2.95 x 10 ^-4^	2.94 x 10 ^-4^
NAS	3.16 x 10 ^-6^	1.58 x 10 ^-5^	5.24 x 10 ^-5^	2.62 x 10 ^-3^	2.60 x 10 ^-3^
AS	6.76 x 10 ^-7^	3.38 x 10 ^-6^	6.91 x 10 ^-5^	3.46 x 10 ^-3^	3.45 x 10 ^-3^

**Figure 7.**
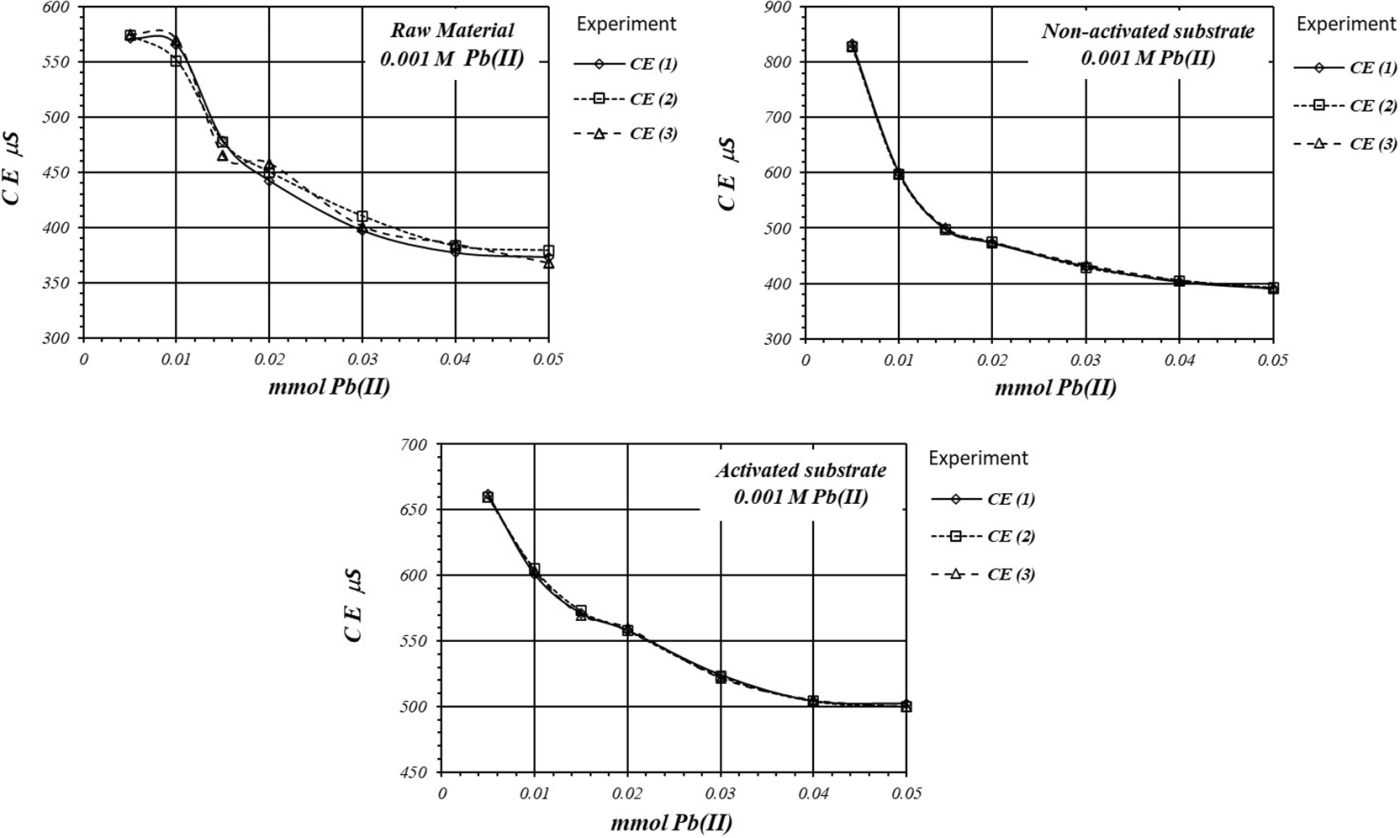
EC variation, by triplicate, as a function of mmol of Pb(II) added to 2 *g* of substrate from a solution 0.001 M Pb(II), for crud material, activated and non-activated substrate.

Figure 7 shows EC, variation, by triplicate, during adsorption reaction of Pb(II) ions on the activate and non-activated substrates, as well as on the raw material, using a 1 *m*M Pb(II) solution. EC in the solution is basically produced by ions in the solution, *i.e.,* CH _3_COO ^-^ and Pb(II) ions, as well as H ^+^ ions produced in the adsorption reaction. As EC decreases, it suggests that Pb(II) ions are adsorbed on the substrate surface, as well as on the raw material. The adsorption reaction produces ion immobilization being unable to make a net contribution to the EC of the solution.

**Figure 8.**
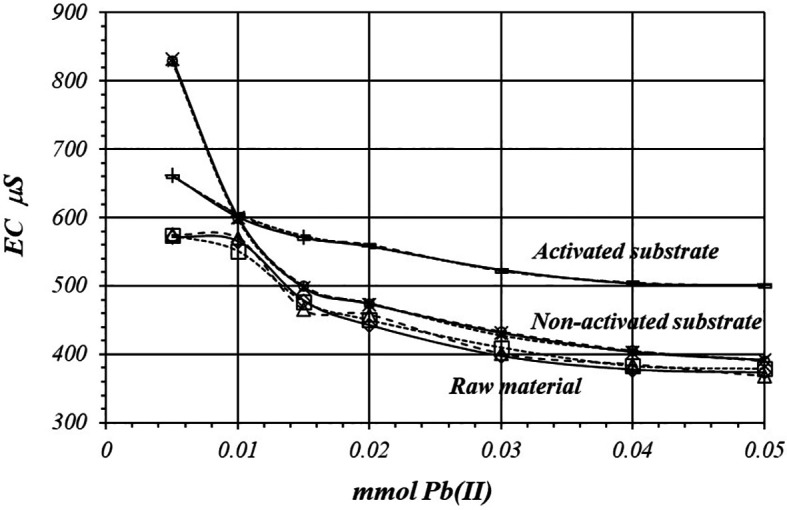
EC variation, by triplicate, as a function of mmol of Pb(II) added to 2 *g* of substrate from a solution 1 *m*M Pb(II), for crud material, activated and non-activated substrate.

Figure 8 shows a comparative view of the EC of the solutions for all three cases when a 1 *m*M Pb(II) solution is used. Although conductivity decrease due to adsorption of Pb(II) ions, solution in contact with activated substrate present greater conductivity due to the highest production of H ^+^ ions during the adsorption reaction. These H ^+^ ions have a net contribution to the EC of the solution producing an increasing of the conductivity in the solution which is in contact with the substrates.

**Figure 9.**
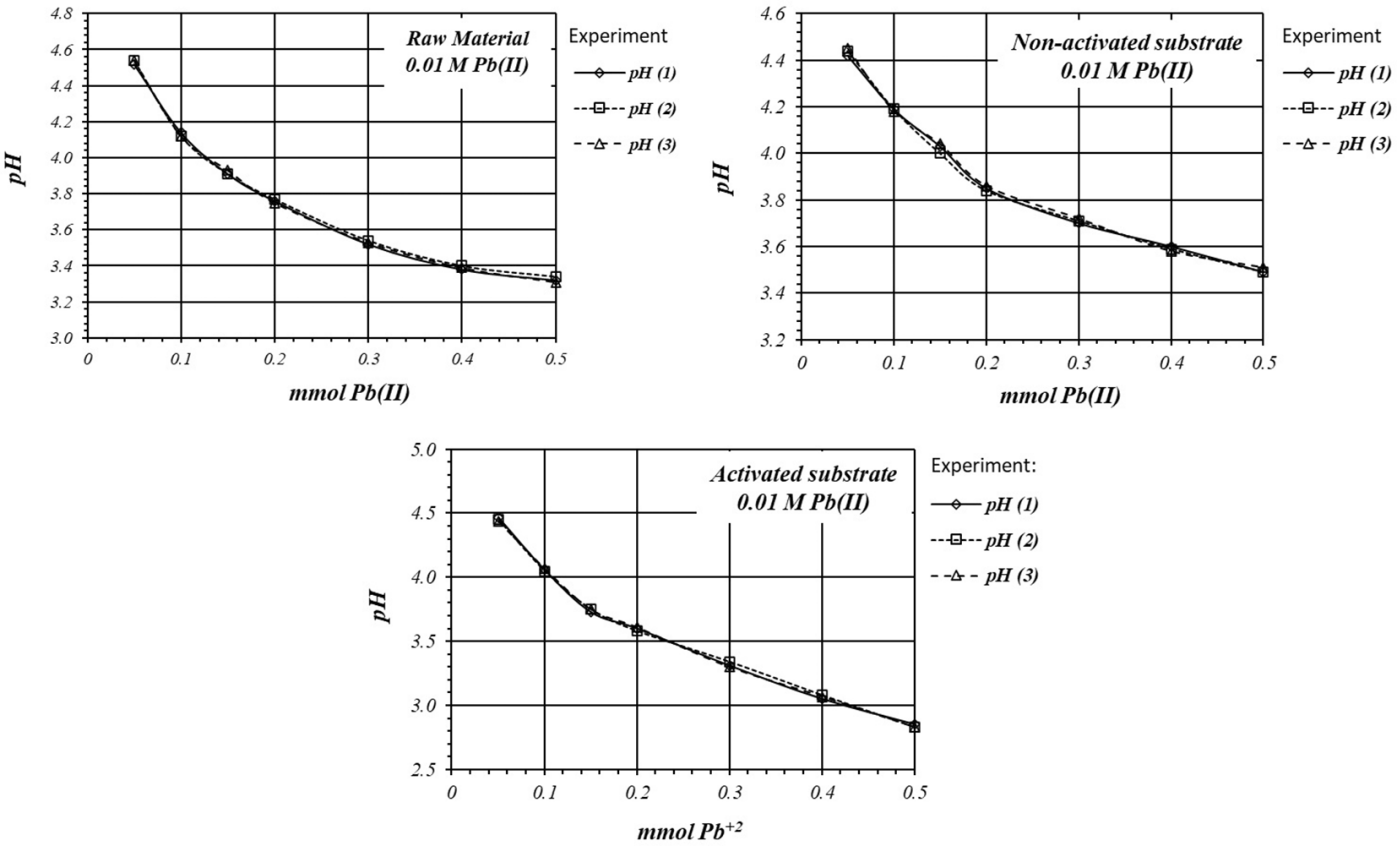
*p*H variation, by triplicate, as a function of mmol of Pb(II) added to 2 *g* of substrate from a 10 *m*M Pb(II) solution, for raw material, and activated and non-activated substrate.

Figure 9 shows *p*H variation, by triplicate trial, during adsorption reaction of Pb(II) ions on activate and non-activated substrates as well as on the raw material, when using a 10 *m*M of Pb(II) ions solution. In line with previous instances, the reaction induces the acidification the solution, a phenomenon concurrently escalating with the rise in Pb(II) ion concentration.

**Figure 10.**
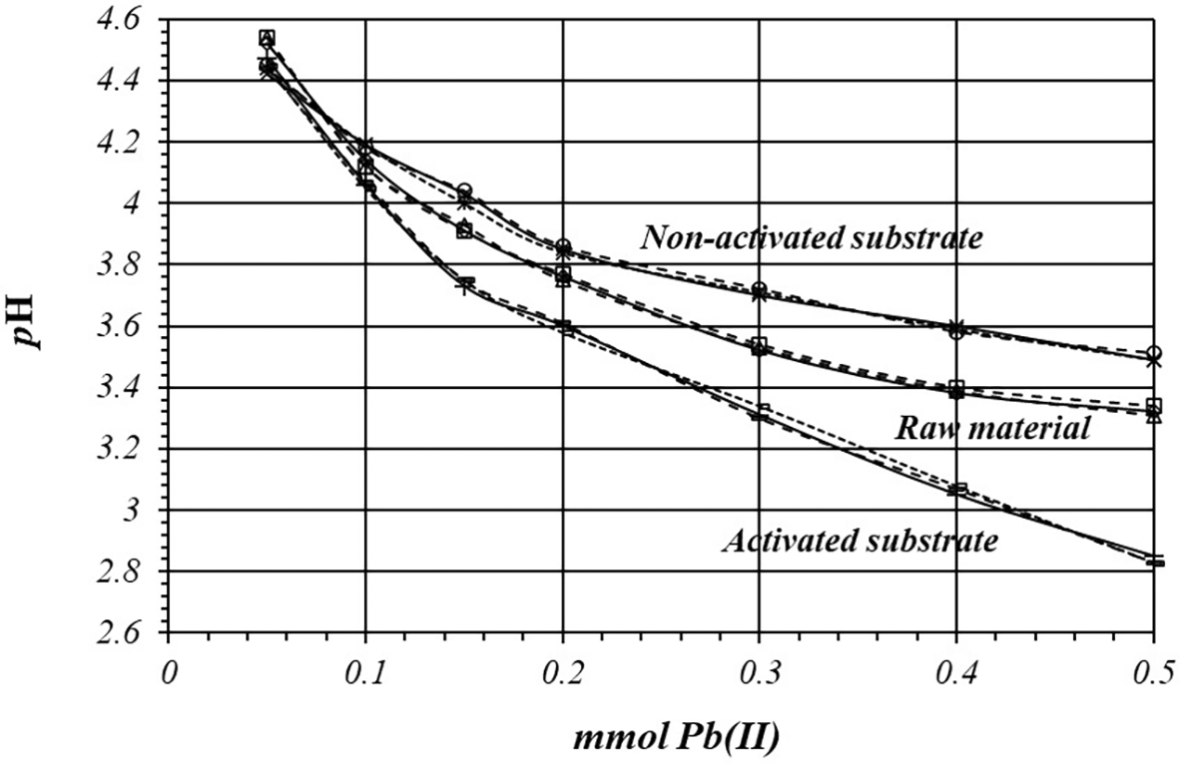
*p*H variation as a function of mmol of Pb(II) added to 2 *g* of substrate from a solution 10 *m*M Pb(II), for crud material, activated and non-activated substrate.

Figure 10 shows comparative *p*H variations during adsorption reaction for all these three cases, when a 10 *m*M of Pb(II) solution is used. Acidification process is more accentuated on the activated substrate, as is expected. The experiments are highly reproducible as in the previous case ( Figure 9), so reaction takes place through the same mechanism in all the replicates.

Table 6 shows initial and final concentrations of H ^+^ ions during the adsorption reaction of Pb(II) ions and the net amount of mmol of H ^+^ ions produced in the whole reaction, when a 10 *m*M Pb(II) solution is used. Adsorption reaction on activated substrate produces 72.2 μmol of H ^+^ ion compared with 15.6 μmol of H ^+^ on non-activated substrate, which is almost five times smaller. Adsorption reaction on raw material produces 23.3 μmol of H ^+^ ion, which is 3 times smaller than in the case of adsorption reaction on the activated substrate. Moreover, this amount of H ^+^ ions is almost 200 times greater than in the former case when used a 1 *m*M Pb(II) solution for adsorption reaction.

**Table 6.** mmol of H ^+^ ion produced during the reaction of adsorption when a 10 *m*M Pb(II) solution is used.

Material	C _i_ H ^+^ mmol/mL	mmol 5 mL	C _f_ H ^+^ mmol/mL	mmol 50 mL	mmol H ^+^ produced
RM	2.95 x 10 ^-5^	1.47 x 10 ^-4^	4.68 x 10 ^-4^	0.0234	0.0233
NAS	3.72 x 10 ^-5^	1.86 x 10 ^-4^	3.16 x 10 ^-4^	0.0158	0.0156
AS	3.55 x 10 ^-5^	1.77 x 10 ^-4^	1.45 x 10 ^-3^	0.0723	0.0722

**Figure 11.**
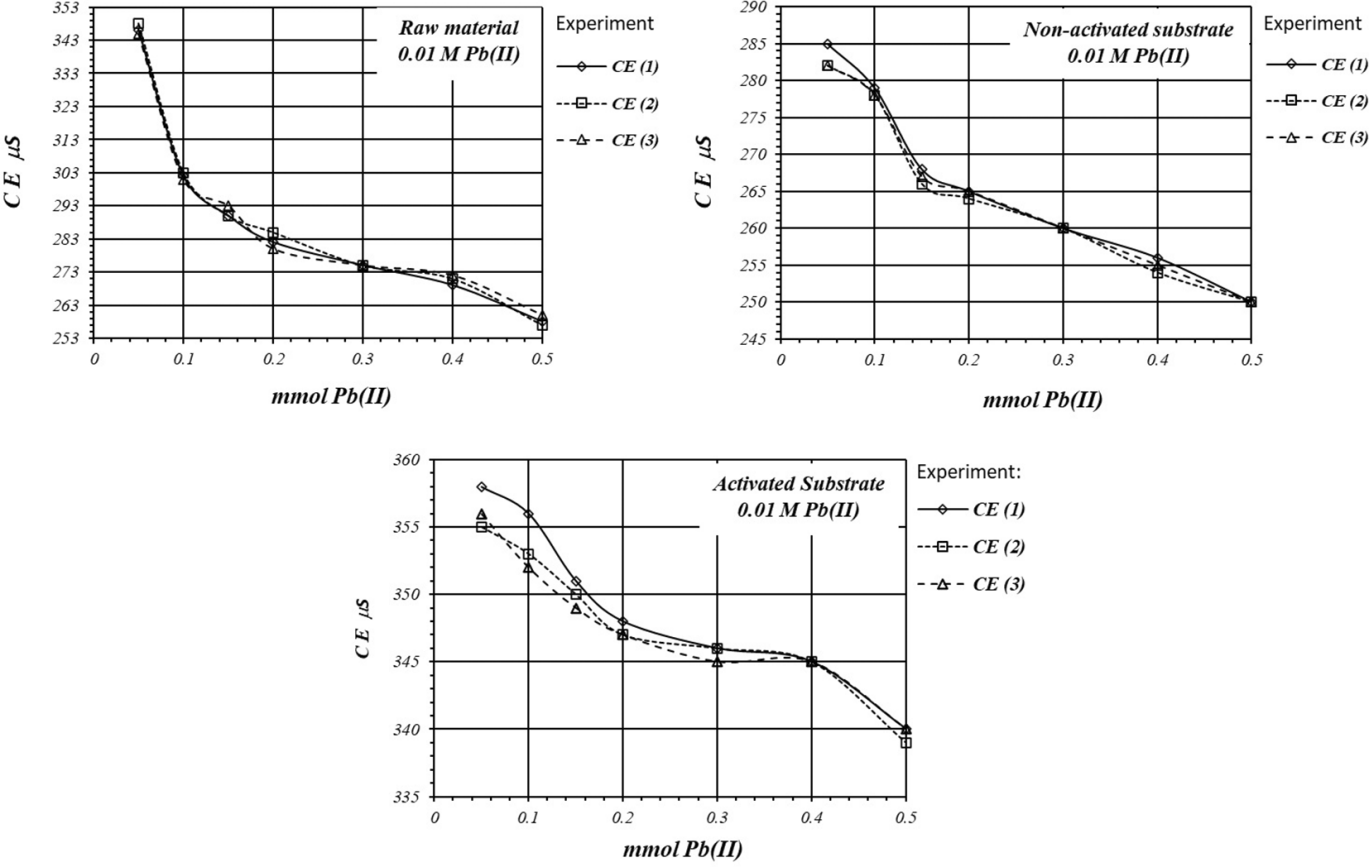
EC variation as a function of mmol of Pb(II) added to 2 *g* of substrate from a solution 10 *m*M Pb(II), for crud material, activated and non-activated substrate.

**Figure 12.**
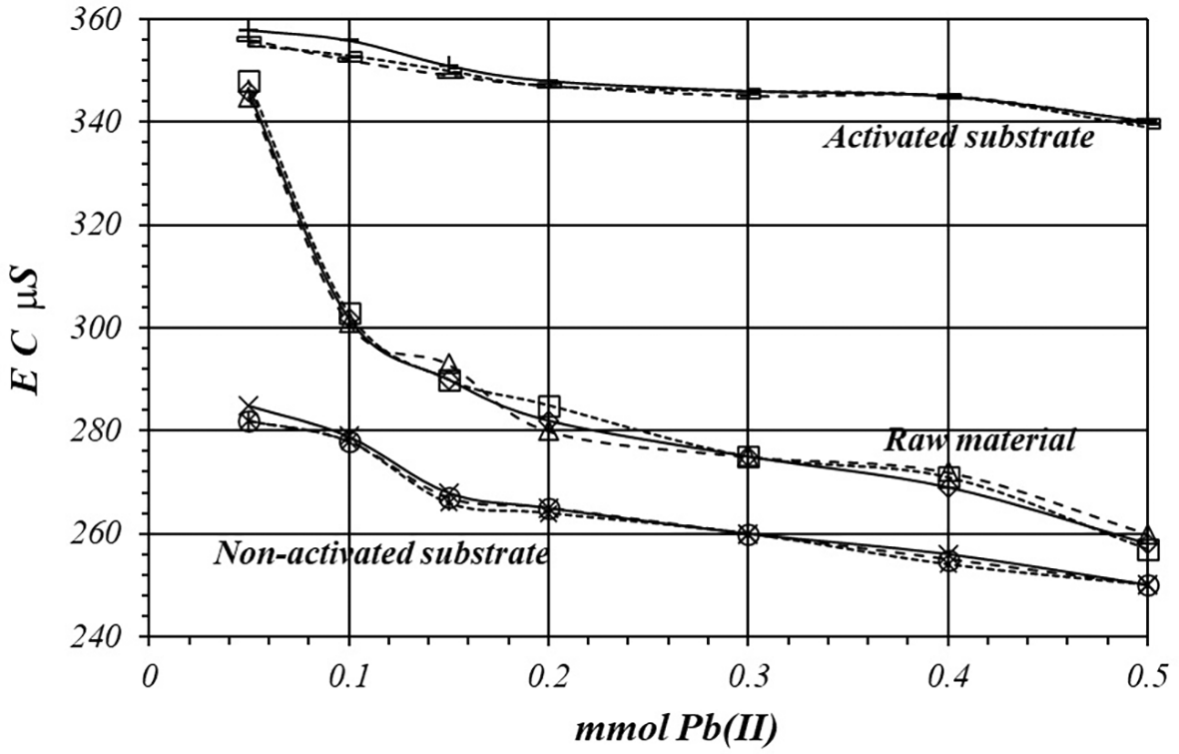
EC variation as a function of mmol of Pb(II) added to 2 *g* of substrate from a 10 *m*M Pb(II) solution, for raw material, activated and non-activated substrate.

Figures 11 and 12 shows EC, variation by triplicate, during adsorption reaction of Pb(II) ions on raw material and non-activated and activates substrates, when a 10 *m*M Pb(II) is used. EC in the solutions decreases, because Pb(II) ions are adsorbed on the substrate’s surfaces, however, the decreasing of EC is less pronounced in the solution in contact with the activated substrate A higher net production of protons rises the EC in the solution. Solutions in contact with the non-activated substrate and the raw material show lower EC values due to the less production of H ^+^ ion during the adsorption reaction.

## Discussion

As pointed out earlier, the amphoteric oxides of Fe, Al, Mn and Ti present on the oxidic substrate surface, which can modify surface charges according to *p*H value. In alkaline media, a deprotonation reaction of the oxides take place, increasing surface negative charge density, while in acid media, surface oxide protonation occur, increasing positive charge density according to equation (5) ^4, 7, 40^:

$$H_{2}O+{\mathrm{FeO}}^{-}\leftarrow \mathrm{OH}-\mathrm{Fe}-\mathrm{OH}\to H+\mathrm{Fe}-{\text{OH}}^{2+}$$ (5)

These materials can be classified as “ *Variable Charge Oxidic Lithologic Materials*”. This particular property can be applied in ionic adsorption processes and exploited in water treatment for water softening, or removing of pollutant chemical species as heavy metals. Literature suggests a mechanism for the adsorption of transitional metals, on these kinds of surfaces, through the formation of an inner sphere complex between metal ion and the oxidic surface, according to the equation (6) ^4, 25, 41^:

$$>{\text{FeOH}}^{-1/2}+{{\mathrm{MH}}_{2}O_{6}}^{+n}\to >\mathrm{Fe}-O-{{\mathrm{MH}}_{2}O_{5}}^{+n-3/2}+H_{3}O^{+}$$ (6)

Such a kind of reaction modifies surface charge by increasing the positive charge density, with the formation of H _3_O ^+^ ion, producing solution acidification. This kind of adsorption is defined as specific adsorption or chemisorption, presenting great tendency to irreversibility. ^4, 5^

Although it does not confirm information about the interaction between Pb(II) ions and calcined substrate surface, an *L*-type isotherm is indicative of great affinity between Pb(II) ions and calcined substrate surfaces. ^36, 37, 41, 42^ It might suggests that adsorption reaction on the activated substrate take place through chemisorption of Pb(II) ions on the oxidic surface. At low equilibrium concentrations, the adsorbed amount increases rapidly, while at high concentrations it decreases, reaching to a saturation zone. The adsorption phenomenon is faster and more intensive on the activated substrate because more favorable sites for adsorption are available on the adsorbent substrate surface, according to reaction (5). Therefore, the probability for the Pb(II) ion adsorption is greater on the activated substrate than on the non-activated substrate.

The flat part of the isotherm ( Figure 1a, *activated substrate*) suggests formation of a saturated monolayer of Pb(II) ions on the surface as is predicted by the Freundlich and Langmuir models and chemisorption should occur on a single monolayer. This type of isotherm, points to the formation of a covalent bond between Pb(II) ions and the substrate surface that is formed by amphoteric metallic oxides as iron, aluminum, titanium and manganese oxides with variable surface charges. ^1, 2, 5, 6^ Similar type of isotherm has been reported in the literature for Cu(II) ions on OLM calcined substrates. ^6, 29, 41– 44^

However, the Langmuir model has its own limitations when used to explain the adsorption process on non-homogeneous surfaces. This model was developed for gas adsorption on homogeneous surfaces; therefore, the model might fail when adsorption take place on irregular surfaces as on the oxidic adsorbent substrate. One of the disadvantages of the Langmuir model is to assume the formation of a monolayer on a homogeneous surface, where all the available sites for the adsorption are equivalent and the ΔH _ad_ is independent of the degree of surface coverage. However, on a non-homogeneous or irregular surface the adsorption sites are non-equivalent and the ΔH _ad_ varies from one place to another. Consequently, those places which led to a more stable bonding are first occupied. The interaction between adsorbed molecules might affect the affinity between the adsorbate and adsorbent, and the greater the surface coverage is, the smaller the ΔH _ad_ is also increases the repulsion between the adsorbed molecules. It might cause the mobility of the molecules through the surface and different places can be occupied. As a result, a physisorbed layers can be formed over the chemisorbed layer. ^35, 36, 40– 42^

On the contrary Freundlich model adapts better to non-homogeneous surfaces, consequently, the adsorption from aqueous phase on non-homogeneous or irregular surfaces like the calcined substrate surface, the Freundlich model fits better. ^2, 35– 37, 41^

On the non-activated substrate Adsorption reaction ( Figure 1 *a*), it seems to run by two different mechanisms. At lower concentrations it follows an *L*-type isotherm, showing much less affinity for Pb(II) ions compared to activated substrate, the smallest negative charge density on the surface is a limiting factor for the adsorption of Pb(II) ion. However, at higher concentrations, adsorption follows a linear model, suggesting a collateral mechanism for the Pb(II) ions adsorption. Most likely acetate ions, being strong base, might act as a deprotonation agent in favor of the adsorption of Pb(II) ions, according to the reaction (7):

$$\text{FeOH}+CH_{3}\mathrm{CO}O^{-}\to \mathrm{Fe}O^{-}+CH_{3}\text{COOH}$$ (7)

Therefore, adsorption of Pb(II) ions take place after oxide deprotonation via acetate ion, according to a multilayer physic adsorption model. After all, acetate ions, being a strong base, might compete in the oxide deprotonation to form molecular acetic acid. However, the acetate effect doesn’t appear on the activated substrate because the oxidic surface is already deprotonate by the previous alkaline attack.

As it was pointed out earlier, non-activated substrate data serves only as a reference to confirm the oxides deprotonation reaction after alkaline treatment on the activated substrate, so non-activated substrate is useless without being chemically treated in alkaline medium.

The *p*H measurements during the adsorption reaction showed a significant acidification along the reaction, which agree with the literature ^4, 5, 38, 40, 43^ about transitional metals chemisorption on amphoteric surface with variable charge. This acidification process became more intense as the concentration of Pb(II) ions in the solution increases. Along with the *L*-type isotherm, the acidification might be interpreted in terms of a covalence between Pb(II) ions and the oxidic surface, into a specific adsorption reaction or chemisorption, according to the model presented in the equation 8 suggest by the literature. ^1, 5, 29, 44^

$$>M-{\mathrm{OH}}^{-0.5}+{{\mathrm{PbH}}_{2}O_{4}}^{+2}\to >M-O-{{\mathrm{PbH}}_{2}O_{3}}^{+0.5}+H_{3}O^{+}$$ (8)

Actually, the new negative charges formed during the alkaline reaction of oxides deprotonation are temporally neutralized by sodium ions, from NaOH solution, which finally will be replaced by Pb(II) ions in the adsorption reaction. EC measurements confirm the acidification process, the highest EC values in the solution in contact with the activated substrate might be interpreted in terms of a highest proton production during the adsorption reaction on the activated substrate.

According to the results, the adsorption reaction is more favorable on the activated substrate where more adsorption active sites are available, due to the alkaline treatment, which increased and homogenize the negative charge density along the substrate surface. The small negative charge density on the non-activated substrate is a limiting factor for the adsorption to occur. Similar results have been reported for the adsorption of heavy metals (Cu, Cd, Zn, As, Ni, Mn, Hg, Cr) on biomaterials, zeolites, polymers, carbonaceous materials, and activated clay, where the increment of the *p*H increases the negatively charged sites because the deprotonation reaction the sorbent surface, favoring adsorption phenomena. ^6, 9, 10, 14, 19, 29, 37, 44^ Therefore, it is expected that other transitional metals can suffer the kind of reaction on these kinds of oxidic substrates, making possible their retention from contaminated waters during a filtration process in a granular media.

For activated substrate, adjustment data by Freundlich and Langmuir models fits well with experimental data; however calculated values are biased from the experimental data 15% for the Freundlich model and 25% for the Langmuir model, most probably because of the lack of homogeneity on the oxidic surface, on the contrary, substrate present a rough and irregular surface. Less homogeneity on the surface means that not all adsorbent positions are equivalent. Therefore, the condition of equivalent adsorption sites is no longer fulfilled. Actually, both models try to explain the same type of isotherm, but Freundlich is an empirical model with less straightening conditions compared to the Langmuir which was developed on valuable ideal theoretical considerations difficult to respect in the case of the calcined substrate.

Adsorption isotherms are expressions that define the energy distribution in the active sites and the heterogeneity of the adsorbent surface. This is why when different types of materials and experimental conditions are used, the isotherm can vary. In general, the Langmuir and Freundlich models have been reported that can describe the adsorption process of Pb(II) using clay modified adsorbents, biomaterials, activated carbon, zeolites and multi walled carbon nanotubes. ^6, 10, 14, 16^

## Conclusion

The work focused on the study of the adsorption of Pb(II) ions on the variable charge oxidic substrate, prepared from a natural oxidic lithologic material by thermal treatment and chemically treated or activated in alkaline medium, in order to create a homogenous negative surface charge density where Pb(II) ions can be adsorbed. Adsorption described by an *L*-type isotherm, show great affinity between Pb(II) ions and the activated surface, adjusted to the Freundlich model. The evidence points to the formation of a covalence between the oxidic surface and Pb(II) ion, with solution acidification. *p*H measurements during the adsorption reaction showed important acidification along the adsorption reaction according with the theoretic model of chemisorption Results suggest that this variable charge oxidic adsorbent substrate have great potential as an alternative technique for water treatment at small and medium scale using granular filtration system. Alkaline and acid treated substrates may be combined to retain cationic and anionic species sequentially. Such kind of system will allow not only water softening but removal of other pollutant as heavy metals, organic matter and important turbidity reducing. The easiness and low price make them suitable to apply in rural media without treating water systems, using a low-cost and reliable adsorption system.

### Ethical considerations

Not applicable.

## Data Availability

Figshare: Adsorption of Pb(II) ions on Variable Charge Oxidic Calcined Substrates with Chemically Modified Surface https://doi.org/10.6084/m9.figshare.22266772.v2. ^39^ This project contains the following underlying data:
• Study Adsorption Pb Freundlich model.xlsx • Study Adsorption Pb Langmuir model.xlsx • Study Adsorption Pb H+ produced.xlsx • Study pH Adsorption Pb.xlsx • Study CE Adsorption Pb.xlsx Data are available under the terms of the Creative Commons Deed License (CC0 1.0 Universal).
