# Supplementary material for: Adsorption of Pb (II) ions on variable charge oxidic calcined substrates with chemically modified surface
Source: F1000Res. 2024 Mar 28;12:747. Originally published 2023 Jun 26. [Version 2] doi: 10.12688/f1000research.132880.2 (PMC10989241; doi:10.12688/f1000research.132880.2)
Supplement: Peer review history

# Reviewer report: Reviewer response for version 2

**DOI:** 10.5256/f1000research.163125.r260344

**Published:** 2024-04-02

**Authors:** Nicolae Dinca

**Affiliations:** 1 Department of Technical and Natural Sciences, Faculty of Food Engineering, Tourism and Environmental Protection, “Aurel Vlaicu” University, Arad, Romania

Thanks, the revised form includes all my requests for improving the work. I have no other comments to make.

Is the work clearly and accurately presented and does it cite the current literature?

Yes

If applicable, is the statistical analysis and its interpretation appropriate?

Yes

Are all the source data underlying the results available to ensure full reproducibility?

Yes

Is the study design appropriate and is the work technically sound?

Yes

Are the conclusions drawn adequately supported by the results?

Yes

Are sufficient details of methods and analysis provided to allow replication by others?

Partly

Reviewer Expertise:

chemistry

I confirm that I have read this submission and believe that I have an appropriate level of expertise to confirm that it is of an acceptable scientific standard.

# Reviewer report: Reviewer response for version 1

**DOI:** 10.5256/f1000research.145832.r196306

**Published:** 2023-11-03

**Authors:** Titus Chinedu Egbosiuba

**Affiliations:** 1 Texas A&M University, College Station, Texas, USA

**F1000 Research** **Review**

 The study reported “Adsorption of Pb (II) ions on variable charge oxidic calcined substrates with chemically modified surface **”. **The authors prepared oxidic calcined and chemically modified substrates for the removal of Pb (II) ions from wastewater. I recommend the reconsideration of the manuscript for approval after the following comments are addressed:

**Comments**

1. The language of the manuscript must be improved by a native English editor. There are a number of grammatical errors.
2. I suggest the addition of continuous line numbering to enhance review effectiveness.
3. The abstract must be summarized and rewritten to briefly capture the background, aim of the study, methodology, key results, and the main conclusion. Additional quantification of the results in the abstract is recommended.
4. In the introduction, describe the hazardous effects of the Pb(II) ions heavy metals. The suggested literatures can guide the authors.
5. I suggest the addition of more treatment techniques such as photocatalysis, adsorption, advanced oxidation process, membrane filtration. Also, each of the mentioned techniques must be referenced. The recommended literature can guide the authors.
6. 2 ^nd^ paragraph: “Amorphous nanoaluminophosphates have also been proved for lead removing from…” I suggest removing be changed to removal. The entire statement must also be rephrased accordingly.
7. How was the OLM chemically characterized and where are the results?
8. Under the preparation of adsorbent, “The lithologic material was crushing…” Crushing or crushed?
9. Why use furnace to dry at 150 ^o^C. Is drying done with furnace or oven?
10. How is oxides formation favored by calcination? Reference please.
11. Kindly remove this statement from the bracket. “(The non-treated...substrate).
12. The suggested literatures can be used to reference the model equations.
13. Be consistent in the use of either Pb(II) ions or Pb ^+2^.
14. I suggest the removal of grid lines from the graphs.
15. Table 1 and 3 shows the Freundlich and Langmuir isotherm parameters, not fitted equations.
16. Merge Table 1 and 2. Evaluate the qe parameter and present in Table 1.
17. Also merge Table 3 and 4.
18. Under pH and electric conductivity study, “….and activates substrates” Is it activates or activated?
19. There is no conclusion at the end of the manuscript, is that the journal format?
20. I recommend the following references must be added to enrich the manuscript ^1^^-^^18^.

Is the work clearly and accurately presented and does it cite the current literature?

Yes

If applicable, is the statistical analysis and its interpretation appropriate?

Yes

Are all the source data underlying the results available to ensure full reproducibility?

Yes

Is the study design appropriate and is the work technically sound?

Yes

Are the conclusions drawn adequately supported by the results?

No

Are sufficient details of methods and analysis provided to allow replication by others?

Yes

Reviewer Expertise:

Materials synthesis for wastewater treatment, Adsorption studies, Kinetic and Isotherm models, Reusability study and the Mechanism of adsorption studies.

I confirm that I have read this submission and believe that I have an appropriate level of expertise to confirm that it is of an acceptable scientific standard, however I have significant reservations, as outlined above.

## References

[1] : Green synthesis of iron oxide nanoparticles by Taguchi design of experiment method for effective adsorption of methylene blue and methyl orange from textile wastewater. *Results in Engineering* .2023;19: 10.1016/j.rineng.2023.101198 10.1016/j.rineng.2023.101198

[2] : Hydrogen production using cocaine metabolite in microbial electrolysis cells. *3 Biotech* .2023;13(11) : 10.1007/s13205-023-03805-7 382 10.1007/s13205-023-03805-7 37920191 PMC10618128

[3] : Photocatalytic Efficiency of Titanium Dioxide for Dyes and Heavy Metals Removal from Wastewater. *Bulletin of Chemical Reaction Engineering & Catalysis* .2022;17(2) : 10.9767/bcrec.17.2.13948.430-450 430-450 10.9767/bcrec.17.2.13948.430-450

[4] : Isotherm, kinetics, thermodynamics, recyclability and mechanism of ultrasonic assisted adsorption of methylene blue and lead (II) ions using green synthesized nickel oxide nanoparticles. *Environmental Nanotechnology, Monitoring & Management* .2023;20: 10.1016/j.enmm.2023.100818 10.1016/j.enmm.2023.100818

[5] : Isotherm, kinetics, thermodynamics and mechanism of metal ions adsorption from electroplating wastewater using treated and functionalized carbon nanotubes. *Journal of Environmental Chemical Engineering* .2023;11(1) : 10.1016/j.jece.2022.109180 10.1016/j.jece.2022.109180

[6] : Ultrasonic enhanced adsorption of methylene blue onto the optimized surface area of activated carbon: Adsorption isotherm, kinetics and thermodynamics. *Chemical Engineering Research and Design* .2020;153: 10.1016/j.cherd.2019.10.016 315-336 10.1016/j.cherd.2019.10.016

[7] : Development of Ag-doped on multi-walled carbon nanotubes for the treatment of fish pond effluent. *Regional Studies in Marine Science* .2023;58: 10.1016/j.rsma.2022.102797 10.1016/j.rsma.2022.102797

[8] : Highly efficient as-synthesized and oxidized multi-walled carbon nanotubes for copper(II) and zinc(II) ion adsorption in a batch and fixed-bed process. *Journal of Materials Research and Technology* .2021;15: 10.1016/j.jmrt.2021.09.094 2848-2872 10.1016/j.jmrt.2021.09.094

[9] : Adsorptive properties of MgO/WO3 nanoadsorbent for selected heavy metals removal from indigenous dyeing wastewater. *Process Safety and Environmental Protection* .2022;162: 10.1016/j.psep.2022.04.057 775-794 10.1016/j.psep.2022.04.057

[10] : Taguchi optimization design of diameter-controlled synthesis of multi walled carbon nanotubes for the adsorption of Pb(II) and Ni(II) from chemical industry wastewater. *Chemosphere* .2021;266: 10.1016/j.chemosphere.2020.128937 128937 10.1016/j.chemosphere.2020.128937 33280844

[11] : Activated multi-walled carbon nanotubes decorated with zero valent nickel nanoparticles for arsenic, cadmium and lead adsorption from wastewater in a batch and continuous flow modes. *J Hazard Mater* .2022;423(Pt B) : 10.1016/j.jhazmat.2021.126993 126993 10.1016/j.jhazmat.2021.126993 34530269

[12] : Enhanced adsorption of As(V) and Mn(VII) from industrial wastewater using multi-walled carbon nanotubes and carboxylated multi-walled carbon nanotubes. *Chemosphere* .2020;254: 10.1016/j.chemosphere.2020.126780 126780 10.1016/j.chemosphere.2020.126780 32353809

[13] : Adsorption of Cr(VI), Ni(II), Fe(II) and Cd(II) ions by KIAgNPs decorated MWCNTs in a batch and fixed bed process. *Sci Rep* .2021;11(1) : 10.1038/s41598-020-79857-z 75 10.1038/s41598-020-79857-z 33420137 PMC7794394

[14] : Industrial Wastewater Treatment.2017; 10.1016/B978-0-12-409548-9.10167-8 23-42 10.1016/B978-0-12-409548-9.10167-8

[15] : Photocatalytic, electrochemical, antibacterial and antioxidant behaviour of carbon-sulphur Co-doped zirconium (IV) oxide nanocomposite. *Cleaner Chemical Engineering* .2022;3: 10.1016/j.clce.2022.100034 10.1016/j.clce.2022.100034

[16] : Photocatalytic and toxicity evaluation of local dyeing wastewater by aluminium/boron doped WO3 nanoparticles. *Journal of Water Process Engineering* .2021;44: 10.1016/j.jwpe.2021.102376 10.1016/j.jwpe.2021.102376

[17] : The porosity of felsic pyroclasts: laboratory validation of field-based approaches. *Bull Volcanol* .2023;85(11) : 10.1007/s00445-023-01679-4 69 10.1007/s00445-023-01679-4 37920291 PMC10618398

[18] : Effective removal of malachite green from local dyeing wastewater using zinc-tungstate based materials. *Heliyon* .2023;9(9) : 10.1016/j.heliyon.2023.e19167 e19167 10.1016/j.heliyon.2023.e19167 37662824 PMC10470254

# Reviewer report: Reviewer response for version 1

**DOI:** 10.5256/f1000research.145832.r196304

**Published:** 2023-11-03

**Authors:** Wei Wang

**Affiliations:** 1 Zhengzhou University, Zhengzhou, Henan, China

This paper presents the adsorption of Pb (II) ions on variable charge oxidic calcined substrates with chemically modified surface. Authors should consider the following observations:

     1. Include some novel findings as in numerical results in abstract.

     2. The quality of the data analysis is not solid enough.

     3. The adsorption mechanism was important and needed to discuss in-depth with characterization support.

     4. Compare the present research with the previous literatures.

Is the work clearly and accurately presented and does it cite the current literature?

Yes

If applicable, is the statistical analysis and its interpretation appropriate?

Partly

Are all the source data underlying the results available to ensure full reproducibility?

Partly

Is the study design appropriate and is the work technically sound?

Yes

Are the conclusions drawn adequately supported by the results?

Yes

Are sufficient details of methods and analysis provided to allow replication by others?

Yes

Reviewer Expertise:

Mineral material; adsorption; catalyst

I confirm that I have read this submission and believe that I have an appropriate level of expertise to confirm that it is of an acceptable scientific standard, however I have significant reservations, as outlined above.

# Reviewer report: Reviewer response for version 1

**DOI:** 10.5256/f1000research.145832.r183567

**Published:** 2023-09-05

**Authors:** Nicolae Dinca

**Affiliations:** 1 Department of Technical and Natural Sciences, Faculty of Food Engineering, Tourism and Environmental Protection, “Aurel Vlaicu” University, Arad, Romania

The work is topical because it scientifically substantiates the possibility of valorizing some indigenous raw materials in the process of purifying residual waters containing Pb ^+2^ resulting from human activity.

 The experimental design described on page 4 above, considers the sample volume as a variable factor and the constant Pb ^+2^ concentration (0.001 M), while the results suggest that the sample volume is constant and the concentration is variable. I believe that the confusing text from Methods Adsorption studies (page 4, lines 6-8) should be corrected.

*Ci* is not defined in the text (page 4, line 8).

 If the values of column **n** in Table 1 are well calculated, then they do not correspond to equations (1) and (2) in the form of the work dated 26 Jun 2023, 12:74. I believe that these equations should be corrected by replacing **n** with **1/n**.

 Fig.1 indicates that at low and medium initial ( *Ci*) Pb ^+2^ concentrations, the adsorption speeds (at 24h) are higher for the activated material.

 On page 6, line 5, *k _1_* and *k _2_* appear which are not defined in the text and do not appear in eqs. (3-4). 

*K _1_* (page 6 line 7) is identical to *k _1_* from line 5?

 Technological perspectives that could complement the conclusions (optional):

- The isotherms in Fig. 1 show that an optimal option in practical applications would be the use of two ion exchange columns linked in series: the first one containing non-activated substrate (cheaper) and the second one containing activated substrate.
- pH and conductivity measurements can be accessible technical options for monitoring the purification process.

Is the work clearly and accurately presented and does it cite the current literature?

Yes

If applicable, is the statistical analysis and its interpretation appropriate?

Yes

Are all the source data underlying the results available to ensure full reproducibility?

Yes

Is the study design appropriate and is the work technically sound?

Yes

Are the conclusions drawn adequately supported by the results?

Yes

Are sufficient details of methods and analysis provided to allow replication by others?

Partly

Reviewer Expertise:

chemistry

I confirm that I have read this submission and believe that I have an appropriate level of expertise to confirm that it is of an acceptable scientific standard, however I have significant reservations, as outlined above.

# Response

**Published:** 2023-09-20

**Authors:** JOSE PRATO MORENO

**Affiliations:** Facultad de Ingeniería, Universidad Nacional de Chimborazo, Riobamba, Chimborazo Province, Ecuador

We thank the reviewer for the importance has given to our research, the time spent correcting the manuscript, their knowledge, experience, and willingness to improve the article.

**Comment 1**: The experimental design described on page 4 above, considers the sample volume as a variable factor and the constant Pb ^+2^ concentration (0.001 M), while the results suggest that the sample volume is constant and the concentration is variable. I believe that the confusing text from Methods Adsorption studies (page 4, lines 6-8) should be corrected.

**Response 1: **Seven samples of 2 g of substrate, activated and non-activates, were treated with increasing aliquots of 5, 10, 15, 20, 25, 30 and 40 mL of a 1 mM Pb ^+2^ solution. Samples are let to stand, in isothermal conditions, during 24 hours, and periodically shacked. So, the number of millimoles in each one of the samples is known.

**Comment 2**: Ci is not defined in the text (page 4, line 8).

**Response 2: **It is corrected, defining Ci in the text, section on adsorption studies.

**Comment 3:** If the values of column n in Table 1 are well calculated, then they do not correspond to equations (1) and (2) in the form of the work dated 26 Jun 2023, 12:74. I believe that these equations should be corrected by replacing n with 1/n.

**Response 3: **“n” values are god, what is wrong is equation 2, where exponential term is not n but 1/n. therefore, equations 1 and 2 become:





**Comment 4**: Fig.1 indicates that at low and medium initial (Ci) Pb ^+2^ concentrations, the adsorption speeds (at 24h) are higher for the activated material.

**Response 4:**

 Taking into account the observation made, the wording has been modified regarding Fig 1, in the results and discussion sections:

**Results:**

**Isotherm graph**

 Figure 1 *a* shows the adsorption isotherms of Pb ^+2^ ions on the activated and non-activated substrates. Although the isotherm profile shows but doesn't confirm information about of the interaction between Pb ^+2^ ions and substrate surface, *L*-type isotherm is associated with great affinity between sorbent and sorbate. At low equilibrium concentrations, the amount adsorbed increases rapidly, while at high concentrations it decreases, getting to a saturation zone. At low concentration non-activated substrate shows less affinity for Pb ^+2^ ions compared to activated substrate, however, at higher concentration the adsorption isotherm become linear increasing Pb ^+2^ adsorption. It might suggest collateral mechanism for the lead ions adsorption.

**Discussion:**

 Figure 1 *a*, shows the isotherm for non-activated substrate. At low concentrations, a similar kind of adsorption but much less intense, showing less affinity for Pb ^+2^ adsorption. However, at higher concentrations, adsorption increases according to a linear model. Most likely acetate ions, being strong base, deprotonates oxides in favor of Pb ^+2^ adsorption, according to the reaction (7)

*FeOH + CH _3_COO¯ → FeO¯+ CH _3_COOH*

 Therefore, adsorption of Pb ^+2^ ions take place after oxide deprotonation via acetate ion, according to a multilayer physic adsorption model. After all, acetate ions, being a strong base, might compete in the oxide deprotonation to form molecular acetic acid.

**Comment 5**: On page 6, line 5, k1 and k2 appear which are not defined in the text and do not appear in eqs. (3-4). K1 (page 6 line 7) is identical to k1 from line 5?.

**Response 5:**

 k _1_ and k _2_ have been defined in the text and equations 3 and 4 have been adjusted, as detailed below:





 In equations 3 and 4, *q _e_* is the amount adsorbed per adsorbent weight unit, *C _eq_* is the equilibrium concentration of adsorbate in solution after adsorption, *k _1_* is the Langmuir constant related to the affinity between adsorbate and adsorbent, and *k _2_* is the adsorption capacity, which represents the maximum amount of adsorbate in a monolayer. The parameters of the isotherm are obtained by plotting C _eq_/q _e_ versus C _eq_, resulting in a straight line with slope equal to *1/(k _1_* * *k*_*2*_ *)* and the intersection equal to 1/k _2_.

 Thank you again for your review.

 Sincerely,

 José G. Prato, Fernando Millán, Marialy Rangel, Andrés Márquez, Luisa Carolina González, Iván Ríos, César García, Carlos Rondón, Enju Wang
